# Aczel–Alsina T-norm based group decision-making technique for the evaluation of electric cars using generalized orthopair fuzzy aggregation information with unknown weights

**DOI:** 10.1016/j.heliyon.2024.e26921

**Published:** 2024-03-01

**Authors:** Nan Zhang, Muhammad Rizwan Khan, Kifayat Ullah, Muhammad Saad, Shi Yin

**Affiliations:** a School of Marxism, Hebei Agricultural University, Baoding, 071001, China; b Department of Mathematics, Riphah International University Lahore, Lahore, 54000, Pakistan; c Department of Applied Mathematics and Statistics, Institute of Space Technology Islamabad, Islamabad, Pakistan; d College of Economics and Management, Hebei Agricultural University, Baoding, 071000, China

## Abstract

Data management and finding precise outcomes from large amounts of information are among the biggest challenges for scientists. The technique of multi-attribute group decision-making (MAGDM) is a valuable tool for investigating fuzzy data precisely. The key objective of the paper is to redefine the q-rung orthopair (q-RO) fuzzy set (FS) (q-ROFS) in the term of interval-valued and proposed new aggregation operators (AOs) based on the Aczel-Alsina (AA) t-norm (TN) and t-conorm (TCN) operations. The AA operational laws are a generalized form of existing TNs and TCNs and give more reliable results because they can fluctuate in their parametric values. The concept of interval-valued enlarges the space of membership degree (MD) and non-membership degree (NMD) for decision-makers. By taking qth power, the interval-valued q-ROFS (IV-q-ROFS) structure. The IV-q-ROFS can handle the uncertainty and vagueness in data, then interval-valued intuitionistic FS (IV-IFS) and interval-valued Pythagorean FS (PyFS) (IV-PyFS) and provide accurate results. The thought of power AOs (PAOs) makes the relationship between weight vectors and reduces the chances of uncertainty in aggregated results. By taking advantage of PAOs, this article is devoted to introducing the interval-valued q-ROF Aczel-Alsina power-weighted averaging (IV-q-ROFAAPWA) and interval-valued q-ROF Aczel-Alsina power-weighted geometric (IV-q-ROFAAPWG) operators. The fundamental axioms of AOs, idempotency, boundedness, and monotonicity, are also discussed. To illustrate the importance of suggested AOs, the real-life problem of electric car selection was solved by applying the MAGDM method using the proposed IV-q-ROFAAPWA and IV-q-ROFAAPWG operators. The comparison of proposed AOs with currently present AOs is also part of the article. We finally constructed solid conclusions.

## 1 Introduction

Decision-making (DM) problems are always considered a hot area in the history of mathematics. In this scenario, many mathematicians present different theories. The crip set theory is an indispensable concept in DM sciences. In the idea of crips set theory, there are only two possible ways to present uncertain information: "Yes: or "No." There is space to give the information in which human options are involved. This is the main drawback of the crisp set theory [1]. Introduced a fuzzy set (FS) to eliminate this limitation. FS can describe human opinion in terms of MD under the range [0,1]. Over time [2], introduced the intuitionistic FS (IFS) by highlighting the deficiencies in FS theory. He also extended FS's rage by adding the idea of NMD in IFS. The range of IFS is [0,1]. In addition, the concept of IFS cannot deal with high-range data, such as if the sum of 0.6 and 0.7 is 1.3. This is a violation of FS theory. So, to overcome this type of problem, PyFS discussed in Ref. [3]. PyFS sum of the square of MD and NMD always lies between the range [0,1] and it is the generalized format of IFS. But the structures of IFS and PyFS are unable to print the assessment information like (0.9,0.6) or (0.7,0.8). To reduce this issue [4], introduced q-ROFS by taking the q power on MD and NMD. The assembly of q-ROFS is considered superior to IFS and PyFS because when we take q is equal to 1 and q is equal to 2, the shape of q-ROFS is turned into the IFS and PyFS, respectively.

The MAGDM algorithm is one of the best procedures to aggregate the information and find suitable alternatives. In literature there are many MAGDM algorithms are defined in various fuzzy environments; for example, An interval-valued IFS for MAGDM problems defined in Ref. [5]. The technique of MAGDM in IFS framework is proposed in Ref. [6], and The solution to the MAGDM problem using a PyFS environment based on Einstein AOs given in Ref. [7]. [8] provided the thought of an interval-valued PyFS in the soft fuzzy environment for finding the solution to MAGDM issues and Zhou and [9] provided the solution to MAGDM issues using the Linguistic PyFS environment. The concept of decision-making science uses the Linguistic PyFS derived in Ref. [10], and MAGDM methodology using the IV-q-ROFS framework for Maclaurin symmetric mean AOs discussed in Ref. [11]**.** [12] presented the impression of the MAGDM problem through interval-valued neutrosophic (IVN) information, and the application of MAGDM based on the interval-valued hesitant Einstein-prioritized AOs discussed in Ref. [13]. [14] proposed the Choquet integral AOs for MAGDM problems and a practical example related to the MAGDM method based on the t-spherical fuzzy (TSF) set (TSFS) structure is provided in Ref. [15]. [16] diagnosed AOs by applying the theory of TSFS for group decision-making.

AOs are the primary tool for the assessment of uncertain and fuzzy information. In the MAGDM problems, many AOs based on TN and TCNs are defined by many researchers, such as the MAGDM method based on the prioritized AA picture fuzzy AOs defined in Ref. [17] and the solution of MAGDM based on Maclaurin symmetric AOs introduced in Ref. [18]. The notion of q-ROF Aczel-Alsina AOs (q-ROFAAAOs) was proposed in Ref. [19], and the idea of Interval-valued IF variable hybrid weighted AOs was proposed in Ref. [20]. [21] proposed power Maclaurin symmetric AOs based on the PyFS framework. The complex TSF PAOs solve the DM problem offered in Ref. [22], while the idea of Picture fuzzy (PF) Maclaurin symmetric AOs is defined in Ref. [23]. Th concept of t-spherical fuzzy (TSF) PAOs was proposed in Ref. [24]. Ullah et al. gave the DM problem solution using interval-valued TSF AOs [25]. The thought of generalized orthopair FS given in reference [26,27] provided the solution for constructing the Fangcang shelter hospital by applying the spherical FS theory.‬‬‬‬‬‬‬‬‬‬‬‬‬‬‬‬‬‬‬‬‬‬‬‬‬‬‬‬‬‬‬**‬‬‬‬‬‬‬‬‬‬‬‬‬‬‬‬‬‬‬‬‬‬‬‬‬‬‬‬‬‬‬‬‬‬‬‬‬‬‬‬‬‬‬‬‬‬‬‬‬‬‬‬‬‬‬‬‬‬‬‬‬‬‬‬‬‬‬‬‬‬** The idea of digitalizing transport systems using the concept of spherical FS was offered in reference [28]. The idea of complex q-ROFS using AA operations was discussed in Ref. [28], and [29] presented the solution of urban transport planning using the concept of decision-making sciences. The decision-making technique is used to find suitable path selection for public transportation, as discussed in Ref. [29].

Presented [30] the thought of triangular norms for fuzzy metric spaces. Mathematicians define many TNs and TCNs to solve MAGDM issues; for example, the AA norm concept was first introduced [31] in 1982. The noticeable feature of AA TN and TCN is the significant priority of the changeability of parameters. The IF soft AOs based on Einstein TN and TCN defined into [32] and IF hybrid AOs proposed in Ref. [33], Einstein geometric AOs based on IFS given in Ref. [34], and AOs depend on Archimedean TCN and TN for TSF defined in Ref. [35]. Entropy-based Hamacher AOs in the IFS framework proposed by Ref. [36], Dombi TN and TCN [37], Einstein TN and TCN [14], and Hamacher TN and TCN [38]. In recent years [32], have extensively studied the features and related aspects of TNs. The Hamacher AOs PyFS is defined in Ref. [39]. The PAOs for IFS for Frank TN and TCN were defined in Ref. [40]. The concept of AA operation for hesitant q-ROFS, defined in Refs. [41,42], discussed the AA laws for complex spherical FS. The concept of Bonfirroni AOs for data aggregation was discussed by Ref. [43], and [44] proposed the methodology for the Parsimonious best-worst technique for the assessment of travel mode.

In literature, many researchers solve car selection problems through the MAGDM technique using multiple fuzzy frameworks, for example, the section on commercial electric vehicles through fuzzy logic presented in Ref. [45] and an application for finding the suitable electric car using spherical fuzzy environment proposed in Ref. [46]. [47] developed the methodology for choosing an electric vehicle with high social acceptance, and [48] suggested the fuzzy analytic hierarchy method for car selection [49]. Presented the undefined TOPSIS method for finding a suitable charging station for electric cars and the idea of solar electric car selection using the fuzzy CPPRAS model given in Ref. [50]. [51] Provided the fuzzy logic-based electric car speed control system, and [52] introduced electric vehicles' environmental and economic impacts [53]. Highlighted the importance of electric cars using fuzzy logic approaches and suitable place selection for charging stations utilizing DM sciences presented in Ref. [54].

The constructed AOs in IV-q-ROFS environments are practical tools for dealing with ambiguous and fuzzy information and utilizing the MAGDM problems. IV-q-ROFS is a generalized framework compared to other present interval-valued IFS and interval-valued PyFS frameworks. For q=1 and q=2 the system of IV-q-ROFS is turned into the interval-valued IFS and PyFS, respectively. So, it is seen that the structure of interval-valued IFS and interval-valued PyFS are the parts IV-q-ROFS. Hence, the motivation for the proposed approach is shown as follows:

1. Developed the notion of IV-q-ROFS and a few operations, then demonstrated their properties.
2. Proposed a few extended IV-q-ROFAAPWA and IV-q-ROFAAPWG operators and provided their fundamental axioms.
3. Developed MAGDM algorithm based on proposed AOs and solved numerical examples.
4. Give sensitivity analysis by changing the values of parameters and discuss the flexibility and superiority of AOs.

The main advantages of the suggested approach are discussed as follows:

The proposed work is based on the interval-values q-rung orthopair fuzzy values (IV-q-ROFVs), giving decision makers more reliability and freedom for better data aggregation. The structure of IV-q-ROFS has the superior format of the IV-IFS and IV-PyFS and can deal with fuzzy information where these sets fail. Also, in the presented approach, we use the concept of PAOs, which provides more accuracy in aggregated results by making the relation between weight vectors of attributes. One more significant factor of the developed technique is AA operations because AA operations give more accuracy in aggregated outcomes and provide more diversity and preciseness in results due to the changeability of the parametric value. Considering the above-discussed feature, we construct the IV-q-ROFAAPWA and IV-q-ROFAAPWG operators and offer a MAGDM algorithm for solving real-life problems. Also, compare with existing methods to check the applicability of the developed approach.

This article is structured as follows: Section 2. discusses some basic definitions to help understand the article. In section 3. we proposed IV-q-ROFAAPWA and IV-q-ROFAAPWG operators using q-ROFS information. The MAGDM procedure is discussed in Section 4. A numerical is offered in Section 5. Sensitivity analysis is provided in Section 6 by changing the parameters. The compression with existing AOs is also specific in Section 7. Section 8 discussed the results and significance of the developed approach. Finally, a conclusion is certain in Section 9.

## 2 Preliminaries

This segment presents some basics of the q-ROFS that are important to understand the developed results.

### 2.1 q-rung orthopair fuzzy set

By using the independent parameter q [4], generalized the concept of IFSs by developing the framework of q-ROFSs. It is a more effective tool than IFSs because the variety of the NMG and MG for fuzzy and unclear data to solve real-life problems is vast.

Definition 1 [4] For a universal set (US) U, the q-ROFS can be denoted as:

$$u=\left\{\kappa \left(ᵯ,ᵰ\right):0\leq {ᵯ}^{q}\left(\kappa \right)+{ᵰ}^{q}\left(\kappa \right)\leq 1,q\in Z^{+}\right\}$$

Then we defined hesitant degree P(x) as (ᵯ,ᵰ),ϰ∈U for q-ROFVs is explained as follows:

$$P\left(x\right)=\sqrt[{q}]{1-\;\left({ᵯ}^{q}\left(\kappa \right)+{ᵰ}^{q}\left(\kappa \right)\right)}$$

Definition 2 [4] Suppose universal set U, the IV-q-ROFS can be defined as:

$$u=\left\{\kappa \left(\left[{ᵯ}^{l},{ᵯ}^{u}\right],\left[{ᵰ}^{l},{ᵰ}^{u}\right]\right):0\leq \left(\left[{\left({ᵯ}^{l}\left(\kappa \right)\right)}^{q}+{\left({ᵯ}^{u}\left(\kappa \right)\right)}^{q}\right]+\left[{\left({ᵰ}^{l}\left(\kappa \right)\right)}^{q}+{\left({ᵰ}^{u}\left(\kappa \right)\right)}^{q}\right]\right)\leq 1,q\in Z^{+}\right\}$$

The pair of hesitancy degree $\left(\left[{ᵯ}^{l},{ᵯ}^{u}\right],\left[{ᵰ}^{u},{ᵰ}^{u}\right]\right),\kappa \in U$ for IV-q-ROFVs is specified as follows:

$$P\left(\kappa \right)=\sqrt[{q}]{1-\left(\left[{\left({ᵯ}^{l}\left(\kappa \right)\right)}^{q}+{\left({ᵯ}^{u}\left(\kappa \right)\right)}^{q}\right]+\left[{\left({ᵰ}^{l}\left(\kappa \right)\right)}^{q}+{\left({ᵰ}^{u}\left(\kappa \right)\right)}^{q}\right]\right)}$$

Definition 3 Suppose the collection of IV-q-ROFVs $\left(\left[{ᵯ}_{i}^{l},{ᵯ}_{i}^{u}\right],\left[{ᵰ}_{i}^{l},{ᵰ}_{i}^{u}\right]\right),\left(i=1,2,\ldots ,n\right)$ the score value (SV) c on the function can be explained below:

$$S\left(c\right)=\left(\left[{\left({ᵯ}_{i}^{l}\left(\kappa \right)\right)}^{q},{\left({ᵯ}_{i}^{u}\left(\kappa \right)\right)}^{q}\right]-\left[{\left({ᵰ}_{i}^{l}\left(\kappa \right)\right)}^{q},{\left({ᵰ}_{i}^{u}\left(\kappa \right)\right)}^{q}\right]\right),\text{SV}\left(c\right)\in \left[-1,1\right]$$

And A(U) be the accuracy value (AVs) is restricted as given by:

$$A\left(c\right)=\left(\left[{\left({ᵯ}_{i}^{l}\left(\kappa \right)\right)}^{q},{\left({ᵯ}_{i}^{u}\left(\kappa \right)\right)}^{q}\right]+\left[{\left({ᵰ}_{i}^{l}\left(\kappa \right)\right)}^{q},{\left({ᵰ}_{i}^{u}\left(\kappa \right)\right)}^{q}\right]\right),A\left(c\right)\in \left[0,1\right]$$

For two IV-q-ROFVs, $C_{1}=\left(\left[{ᵯ}_{1}^{l},{ᵯ}_{1}^{u}\right],\left[{ᵰ}_{1}^{l},{ᵰ}_{1}^{u}\right]\right)$ and $C_{2}=\left(\left[{ᵯ}_{2}^{l},{ᵯ}_{2}^{u}\right],\left[{ᵰ}_{2}^{l},{ᵰ}_{2}^{u}\right]\right)$ the SV of $U_{i}$ and A(c) is the AVs of $U_{i}$ then $c_{1}>c_{2}$ where the notion $\prime \prime {>}^{\prime \prime }$ represents ‘’more suitable’’ if also $S\left(c_{1}\right)>S\left(c_{2}\right)$ or $S\left(c_{1}\right)=S\left(c_{2}\right)$ and $A\left(c_{1}\right)>A\left(c_{2}\right)$ holds.

Definition 4 [55] For two IV-q-ROFVs, $a=\left(\left[{ᵯ}_{a}^{l},{ᵯ}_{a}^{u}\right],\left[{ᵰ}_{a}^{l},{ᵰ}_{a}^{u}\right]\right)$ and $b=\left(\left[{ᵯ}_{b}^{l},{ᵯ}_{b}^{u}\right],\left[{ᵰ}_{b}^{l},{ᵰ}_{b}^{u}\right]\right)$ the distances between a and b can explained below in Eqution 1.

(1) $$d\left(a,b\right)=\frac{\left|\left[\left({ᵯ}_{a}^{l}-\;{ᵯ}_{b}^{l}\right)+\left({ᵯ}_{a}^{u}-\;{ᵯ}_{b}^{u}\right)\right]\right|+\left|\left[\left({ᵰ}_{a}^{l}-\;{ᵰ}_{b}^{l}\right)+\left({ᵰ}_{a}^{u}-\;{ᵰ}_{b}^{u}\right)\right]\right|}{2}$$

Definition 5 The PAO was proposed by Yager [44], and it is explained in Eqution 2.

(2) $$\text{PA}\left(a_{1},a_{2},\ldots ,a_{n}\right)=\begin{array}{c} n\;⊕\\ i=1 \end{array}\frac{\sum_{i=1}^{n}\left(1+A\left(\beta_{i}\right)\right).\beta_{i}}{\sum_{i=1}^{n}\left(1+A\left(\beta_{i}\right)\right)}$$

Where Eqution 3. discussed the support values of the PAOs.

(3) $$A\left(b_{i}\right)=\sum_{i=1}^{n}\text{Su}p\left(\beta_{i},\beta_{j}\right)$$

and $\text{Su}p\left(\beta_{i},\beta_{j}\right)$ states the consideration of $\beta_{i}$ and $\beta_{j}$ and it must follow the following situations:

a. $\text{Su}p\left(\beta_{i},\beta_{j}\right)\in \left[0,1\right]$
b. $\text{Su}p\left(\beta_{i},\beta_{j}\right)=\text{Su}p\left(\beta_{j},\beta_{i}\right)$
c. $\text{Su}p\left(\beta_{i},\beta_{j}\right)\geq 2.\;\mathrm{Su}p\left(\beta_{k},\beta_{l}\right)\;\mathrm{if}\;\left|\left(\beta_{i},-\beta_{j}\right)\right|<\left|\beta_{k}-\;\beta_{l}\right|$

To make the linked values emphasize and support one another, the connection of aggregate values and their weight vector (WV) of PAOs will depend on their justifications.

Aczel Alsina TN and TCN

Definition 6 [30], Consider TN be the function $z:{\left[0,1\right]}^{2}\to \left[0,1\right]$, and consider triple fuzzy values (FV) for instance τ,χ,Ϸϵ[0,1], fulfill the next properties, as symmetric ¥(τ,χ)=¥(χ,τ); monotonic ¥(τ,χ)≤¥(χ,Ϸ) then χ≤Ϸ and; associative ¥(τ,¥(χ,Ϸ))=¥(¥(τ,χ),Ϸ); then condition ¥(τ,1)=τ holds for identity.

Examples 1 For example, TN can be defined as the product of two values is ${¥}_{Ϸ}\left(ŧ,ņ\right)=ŧ.ņ$. The minimum of TN is written as ${¥}_{M}\left(ŧ,ņ\right)=\min\left(ŧ.ņ\right)$. The Lukasiewicz TNs written as ${¥}_{L}\left(ŧ,ņ\right)-\min\left(ŧ+ņ-\left[1,0\right]\right)$; also, Drastic TNs are explained in Equation (4). as follows:

(4) $${¥}_{D}=\left(ŧ,ņ\right)=\left\{ \;\begin{array}{c} ŧ\;\text{if}\;ņ=1\\ ņ\;\text{if}\;ŧ=1\\ 0\;\text{otherwise} \end{array}\right.\;\forall \;ŧ,ņ\in \left[0,1\right]$$

Definition 7 [56] Define the TCN $Ⱨ:{\left[0,1\right]}^{2}\to \left[0,1\right]$ and suppose three FVs ŧ,ņ,Ϸ be appropriate form [0,1] and fulfill the next properties, as for symmetric Ⱨ(ŧ,ņ)=Ⱨ(ņ,ŧ); monotonic Ⱨ(ŧ,ņ)≤Ⱨ(ņ,ŧ) if ņ≤Ϸ and ŧ≤ņ; associative Ⱨ(ŧ,Ⱨ(ņ,Ϸ))=Ⱨ(Ⱨ(ŧ,ņ),Ϸ); also, The definition of the zero identity condition is Ⱨ(ŧ,0)=ŧ.

Examples 2 For example, a few TNs, like the probabilistic addition of TCN, can be explained by way of ${Ⱨ}_{Ϸ}\left(ŧ,ņ\right)=ŧ+ņ-ŧņ$; Maximum TCN defined by way of ${Ⱨ}_{M}\left(ŧ,ņ\right)=\max\left(ŧ,ņ\right)$. The Lukasiewicz TCNs are defined as follows ${Ⱨ}_{L}\left(ŧ,ņ\right)-\min\;\left(ŧ+ņ-1\right)$; and the Drastic TCNs are explained in Equation (5). as follows:

(5) $${Ⱨ}_{D}=\left(ŧ,ņ\right)=\left\{ \;\begin{array}{c} ŧ\;\text{if}\;ņ=0\\ ņ\;\text{if}\;ŧ=0\\ 0\;\text{otherwise} \end{array}\right.\;\forall \;ŧ,ņ\in \left[0,1\right]$$

[31], When T is TN and Ⱨ is TCN, the next satisfied:

I. Ⱨ(ŧ,ņ)≥max{ŧ,ņ}forallŧ,ņ∈[0,1]
II. Ⱨ(ŧ,ņ)≥min{ŧ,ņ}forallŧ,ņ∈[0,1]

Definition 8 Aczel et al. [31] developed the concept of TNs and TCNs in the early 1980s,

The AATN $\left({¥}_{A}^{Л}\;\right)$ and AATCN $\left({Ⱨ}_{A}^{Л}\;\right)$ can explained below in Equation (6). and Equation (7). as follows:

(6) $$\left(z_{A}^{Л}\;\right)=\left\{ \begin{array}{c} z_{D}\left(ŧ,ņ\right)\;\text{if}\;Л=0\\ \min\left(ŧ,ņ\right)\;\text{if}\;Л=\infty \\ e^{-{\left({\left(-\ln\;ŧ\right)}^{Л}+{\left(-\ln\left(ņ\right)\right)}^{Л}\right)}^{\frac{1}{Л}}}\;\text{otherwise} \end{array}\right.$$

(7) $$\left({Ⱨ}_{A}^{Л}\;\right)=\left\{ \begin{array}{c} {Ⱨ}_{D}\left(ŧ,ņ\right)\;\text{if}\;Л=0\\ \max\left(r,s\right)\;\text{if}\;Л=\infty \\ e^{-{\left({\left(-\ln\left(1-ŧ\right)\right)}^{Л}+{\left(-\ln\left(1-ņ\right)\right)}^{Л}\right)}^{\frac{1}{Л}}}\;\text{otherwise} \end{array}\right.$$

A few important basic facts: ${¥}_{A}^{0}={¥}_{D},{¥}_{A}^{1}={¥}_{P},{¥}_{A}^{\infty }=\min,{Ⱨ}_{A}^{0}={Ⱨ}_{D},{Ⱨ}_{A}^{1}={Ⱨ}_{P},{Ⱨ}_{A}^{\infty }=\max\text{,}$ and for all Л taken from [0,1], the ${Ⱨ}_{A}^{¥}$ and TN ${¥}_{A}^{Л}$ are dual times one another. The AATNs and AATCNs devise an inverse link with each other as we steadily decrease AATCNs and increase the value of AATNs.

Definition 9 [4] For q-ROFVS, operational rules can be defined as listed below:

For q-ROFVs $U_{1}=\left({ᵯ}_{1},{ᵰ}_{1}\right)$ and $U_{2}=\left({ᵯ}_{2},{ᵰ}_{2}\right)$ and for κ>0,

1. $U_{1}⊕U_{2}=\left(\sqrt[{q}]{{ᵯ}_{1}^{q}+{ᵯ}_{2}^{q}-{ᵯ}_{1}^{q}.{ᵯ}_{2}^{q}},{ᵰ}_{1}.{ᵰ}_{2}\right)$
2. $U_{1}⊗U_{2}=\left(\;{ᵯ}_{1}.{ᵯ}_{2},\sqrt[{q}]{{ᵰ}_{1}^{q}+{ᵰ}_{2}^{q}-{ᵰ}_{1}^{q}.{ᵰ}_{2}^{q}}\right)$
3. $\kappa .U=\left(\sqrt[{q}]{1-{\left(1-{ᵯ}^{q}\right)}^{\kappa }},{ᵰ}^{\kappa }\;\right)$
4. $U^{\kappa }=\left({ᵯ}^{\kappa },\sqrt[{q}]{1-{\left(1-{ᵰ}^{q}\right)}^{\kappa }}\right)$

#### 2.1.1 Aczel Alsina operatinal laws for IV-q-ROFVs

This section develops the Aczel-Alsina [24] operating laws using IV-q-ROFVs. Here, we further discuss some hypotheses that are essentially connected.

Definition 10 [57] For two IV-q-ROFVs $U_{1}=\left(\left[{ᵯ}_{1}^{l},{ᵯ}_{1}^{u}\right],\left[{ᵰ}_{1}^{l},{ᵰ}_{1}^{u}\right]\right)$ and $U_{2}=\left(\left[{ᵯ}_{2}^{l},{ᵯ}_{2}^{u}\right],\left[{ᵰ}_{2}^{l},{ᵰ}_{2}^{u}\right]\right)$ and the idea z and Ⱨ to denote the AATNs and AATCNs. So the intersection and union marked by QP respectively and IV-q-ROFVs can explained below:

$$U_{1}⊗U_{2}=\left(\;{¥}_{A}\left\{\left[{ᵯ}_{1}^{l},{ᵯ}_{1}^{u}\right],\left[{ᵯ}_{2}^{l},{ᵯ}_{2}^{u}\right]\right\},{Ⱨ}_{A}\left\{\left[{ᵰ}_{1}^{l},{ᵰ}_{1}^{u}\right],\left[{ᵰ}_{2}^{l},{ᵰ}_{2}^{u}\right]\right\}\right)$$

$$U_{1}⨁U_{2}=\left(\;{Ⱨ}_{A}\left\{\left[{ᵰ}_{1}^{l},{ᵰ}_{1}^{u}\right],\left[{ᵰ}_{2}^{l},{ᵰ}_{2}^{u}\right]\right\},z_{A}\left\{\left[{ᵯ}_{1}^{l},{ᵯ}_{1}^{u}\right],\left[{ᵯ}_{2}^{l},{ᵯ}_{2}^{u}\right]\right\}\right)$$

Definition 11 [57] Let $U=\left(\left[{ᵯ}^{l},{ᵯ}^{u}\right],\left[{ᵰ}^{l},{ᵰ}^{u}\right]\right)$, ${U_{1}=\left(\left[{ᵯ}_{1}^{l},{ᵯ}_{1}^{u}\right],\left[{ᵰ}_{1}^{l},{ᵰ}_{1}^{u}\right]\right)\;\text{and}\;U_{2}=\left(\left[{ᵯ}_{2}^{l},{ᵯ}_{2}^{u}\right],\left[{ᵰ}_{2}^{l},{ᵰ}_{2}^{u}\right]\right)}_{\mu_{2}}$ be IV-q-ROFVs, in this N≥1 and Л≥0.

i $U_{1}⨁U_{2}=\left(\begin{array}{c} \left[\sqrt[{q}]{1-e^{{-\left({\left(-\ln\left(1-{\left({ᵯ}_{U_{1}}^{l}\right)}^{q}\right)\right)}^{Ή}+{\left(-\ln\left(1-{\left({ᵯ}_{U_{2}}^{l}\right)}^{q}\right)\right)}^{Ή}\;\right)}^{\frac{1}{Ή}}}},\sqrt[{q}]{1-e^{{-\left({\left(-\ln\left(1-{\left({ᵯ}_{U_{1}}^{u}\right)}^{q}\right)\right)}^{Ή}+{\left(-\ln\left(1-{\left({ᵯ}_{U_{2}}^{u}\right)}^{q}\right)\right)}^{Ή}\;\right)}^{\frac{1}{Ή}}}}\right],\\ \left[e^{{-\left({\left(-\ln\left({ᵰ}_{U_{1}}^{l}\right)\right)}^{Ή}+{\left(-\ln\left({ᵰ}_{U_{2}}^{l}\right)\right)}^{Ή}\;\right)}^{\frac{1}{Ή}}},e^{{-\left({\left(-\ln\left({ᵰ}_{U_{1}}^{u}\right)\right)}^{Ή}+{\left(-\ln\left({ᵰ}_{U_{2}}^{u}\right)\right)}^{Ή}\;\right)}^{\frac{1}{Ή}}}\right] \end{array}\right)$
ii ${ᵯ}_{U_{1}}⊗{ᵯ}_{U_{2}}=\left(\begin{array}{c} \left[e^{{-\left({\left(-\ln\left({ᵯ}_{U_{1}}^{l}\right)\right)}^{Ή}+{\left(-\ln\left({ᵯ}_{U_{2}}^{l}\right)\right)}^{Ή}\;\right)}^{\frac{1}{Ή}\;},}e^{{-\left({\left(-\ln\left({ᵯ}_{U_{1}}^{u}\right)\right)}^{Ή}+{\left(-\ln\left({ᵯ}_{U_{2}}^{u}\right)\right)}^{Ή}\;\right)}^{\frac{1}{Ή}\;},}\right],\\ \left[\sqrt[{q}]{1-e^{{-\left({\left(-\ln\left(1-{\left({ᵰ}_{U_{1}}^{l}\right)}^{q}\right)\right)}^{Ή}+{\left(-\ln\left(1-{\left({ᵰ}_{U_{2}}^{l}\right)}^{q}\right)\right)}^{Ή}\;\right)}^{\frac{1}{Ή}\;},}}\sqrt[{q}]{1-e^{{-\left({\left(-\ln\left(1-{\left({ᵰ}_{U_{1}}^{u}\right)}^{q}\right)\right)}^{Ή}+{\left(-\ln\left(1-{\left({ᵰ}_{U_{2}}^{u}\right)}^{q}\right)\right)}^{Ή}\;\right)}^{\frac{1}{Ή}\;},}}\right] \end{array}\right)$
iii $ЛU=\left(\left[\sqrt[{q}]{1-e^{{-\left(Л{\left(-\ln\;\left(1-{\left({ᵯ}_{U}^{l}\right)}^{q}\right)\right)}^{Ή}\;\right)}^{\frac{1}{Ή}\;}}},\sqrt[{q}]{1-e^{{-\left(Л{\left(-\ln\;\left(1-{\left({ᵯ}_{U}^{u}\right)}^{q}\right)\right)}^{Ή}\;\right)}^{\frac{1}{Ή}\;}}}\right],\left[e^{{-\left(Л{\left(-\ln\left({ᵰ}_{U}^{l}\right)\right)}^{Ή}\;\right)}^{\frac{1}{Ή}}},e^{{-\left(Л{\left(-\ln\left({ᵰ}_{U}^{u}\right)\right)}^{Ή}\;\right)}^{\frac{1}{Ή}}}\right]\right)$
iv $U^{Л}=\left(\left[e^{{-\left(Л{\left(-\ln\left({ᵯ}_{U}^{l}\right)\right)}^{Ή}\;\right)}^{\frac{1}{Ή}}},e^{{-\left(Л{\left(-\ln\left({ᵯ}_{U}^{u}\right)\right)}^{Ή}\;\right)}^{\frac{1}{Ή}}}\right],\left[\sqrt[{q}]{1-e^{{-\left(Л{\left(-\ln\;\left(1-{\left({ᵰ}_{U}^{l}\right)}^{q}\right)\right)}^{Ή}\;\right)}^{\frac{1}{Ή}}}},\sqrt[{q}]{1-e^{{-\left(Л{\left(-\ln\;\left(1-{\left({ᵰ}_{U}^{u}\right)}^{q}\right)\right)}^{Ή}\;\right)}^{\frac{1}{Ή}}}}\right]\right)$

## 3 IV-q-ROF power aggregation operators

Built on the operational guidelines for IV-q-ROFVs connected to Aczel-Alsina operations provided in part 3, this part discusses IV-q-ROFAAPWA and IV-q-ROFAAPWG aggregation operators.

Definition 12 For assembly of IV-q-ROFVs ${Ґ}_{i}=\left(\left[{\left({ᵯ}_{i}^{l}\right)}^{q},{\left({ᵯ}_{i}^{u}\right)}^{q}\right],\left[{\left({ᵰ}_{i}^{l}\right)}^{q},{\left({ᵰ}_{i}^{u}\right)}^{q}\right]\right)$ and (i=1,2,…,n), and IV-$q-\text{ROFAAPWA}:\gamma^{n}\to \gamma$, discussed in Equation (8). as follows:

(8) $$q-\text{ROFAAPWA}\left(\beta_{1},\beta_{2},\ldots ,\beta_{n}\right)=\begin{array}{c} n\;⊕\\ i=1 \end{array}\frac{\left(\left(1+A\left(\beta_{i}\right)\right)\right)}{\sum_{i=1}^{n}\left(1+A\left(\beta_{i}\right)\right)}.\beta_{i}$$

In this γ can be a collection of all IV-q-ROFVs and $A\left(\beta_{i}\right)=\sum_{\;j=1,j\neq i}^{n}\mathrm{Sup}\left(\beta_{i},\beta_{j}\right)\text{,}$ So IV-q-ROFPA is called the IV-q-ROFAAP aggrigation operator. On behalf of suitability, study

$$\xi_{i}=\frac{1+A\left(\beta_{i}\right)}{\sum_{i=1}^{n}\left(1+A\left(\beta_{i}\right)\right)}$$

Then, Eq. (1) will become

$$\text{IV}-q-\text{ROFAAPWA}\left(\beta_{1},\beta_{2},\ldots ,\beta_{n}\right)=\begin{array}{c} n\;⊕\\ i=1 \end{array}\xi_{i}.\beta_{i}$$

Theorem 1 *For IV-q-ROFVs*${Ґ}_{i}=\left(\left[{\left({ᵯ}_{i}^{l}\right)}^{q},{\left({ᵯ}_{i}^{u}\right)}^{q}\right],\left[{\left({ᵰ}_{i}^{l}\right)}^{q},{\left({ᵰ}_{i}^{u}\right)}^{q}\right]\right)$*in this*i=1,2,3,…,n*so the calculating outcome is also a IV-q-ROFVs through*Definition 8, *is also a IV-q-ROFV*. *The AO can be shown in* Equation (9). *as follows*:

(9) $$\text{IV}-q-\text{ROFAAPWA}\left({Ґ}_{1},{Ґ}_{2},{Ґ}_{3},\ldots ,{Ґ}_{n}\right)=\begin{array}{c} n\;⊕\\ \varpi =1 \end{array}\left({Ґ}_{i}\varpi_{i}\right)=\left(\begin{array}{c} \left[\sqrt[{q}]{1-e^{-{\left(\sum_{i=1}^{n}{\xi_{i}\left(-\ln\left(1-{\left({ᵯ}_{{Ґ}_{i}}^{l}\right)}^{q}\right)\right)}^{Ή}\right)}^{\frac{1}{Ή}}}},\sqrt[{q}]{1-e^{-{\left(\sum_{i=1}^{n}{\xi_{i}\left(-\ln\left(1-{\left({ᵯ}_{{Ґ}_{i}}^{u}\right)}^{q}\right)\right)}^{Ή}\right)}^{\frac{1}{Ή}}}}\right],\\ \left[e^{-{\left(\sum_{i=1}^{n}{\xi_{i}\left(-\ln\left({ᵰ}_{{Ґ}_{i}}^{l}\right)\right)}^{Ή}\right)}^{\frac{1}{Ή}}},e^{-{\left(\sum_{i=1}^{n}{\xi_{i}\left(-\ln\left({ᵰ}_{{Ґ}_{i}}^{u}\right)\right)}^{Ή}\right)}^{\frac{1}{Ή}}}\right] \end{array}\right)$$

*Where* $\xi_{i}$ *and* i=1,2,…,n *is the set of included weights such that*

$$\xi_{i}=\frac{\varpi_{i}\left(1+A\left(\beta_{i}\right)\right)}{\sum_{i=1}^{n}\varpi_{i}\left(1+A\left(\beta_{i}\right)\right)}$$

and $\xi_{i}>0,\sum_{i=1}^{n}\xi_{i}=1$.

Proof: To satisfy that the theorem is valid for n=2, we apply a mathematical induction technique involving the AA operation explained in Definition 8.

$$\varpi_{1}{Ґ}_{1}=\left(\left[\sqrt[{q}]{1-e^{-{\left({\xi_{1}\left(-\ln\left(1-{ᵯ}_{{Ґ}_{1}}^{l}\right)\right)}^{Ή}\right)}^{\frac{1}{Ή}}}},\sqrt[{q}]{1-e^{-{\left({\xi_{1}\left(-\ln\left(1-{ᵯ}_{{Ґ}_{1}}^{u}\right)\right)}^{Ή}\right)}^{\frac{1}{Ή}}}}\right],\left[e^{-{\left({\xi_{1}\left(-\ln\left({ᵰ}_{{Ґ}_{1}}^{l}\right)\right)}^{Ή}\right)}^{\frac{1}{Ή}}},e^{-{\left({\xi_{1}\left(-\ln\left({ᵰ}_{{Ґ}_{1}}^{u}\right)\right)}^{Ή}\right)}^{\frac{1}{Ή}}}\right]\right)$$

$$\varpi_{2}{Ґ}_{2}=\left(\left[\sqrt[{q}]{1-e^{-{\left({\xi_{1}\left(-\ln\left(1-{ᵯ}_{{Ґ}_{2}}^{l}\right)\right)}^{Ή}\right)}^{\frac{1}{Ή}}}},\sqrt[{q}]{1-e^{-{\left({\xi_{1}\left(-\ln\left(1-{ᵯ}_{{Ґ}_{2}}^{u}\right)\right)}^{Ή}\right)}^{\frac{1}{Ή}}}}\right],\left[e^{-{\left({\xi_{1}\left(-\ln\left({ᵰ}_{{Ґ}_{2}}^{l}\right)\right)}^{Ή}\right)}^{\frac{1}{Ή}}},e^{-{\left({\xi_{1}\left(-\ln\left({ᵰ}_{{Ґ}_{2}}^{u}\right)\right)}^{Ή}\right)}^{\frac{1}{Ή}}}\right]\right)$$

*Consider*$r=\left[\sqrt[{q}]{1-e^{-{\left({\xi_{1}\left(-\ln\left(1-{ᵯ}_{{Ґ}_{1}}^{l}\right)\right)}^{Ή}\right)}^{\frac{1}{Ή}}}},\sqrt[{q}]{1-e^{-{\left({\xi_{1}\left(-\ln\left(1-{ᵯ}_{{Ґ}_{1}}^{u}\right)\right)}^{Ή}\right)}^{\frac{1}{Ή}}}}\right],\left[e^{-{\left({\xi_{1}\left(-\ln\left({ᵰ}_{{Ґ}_{1}}^{l}\right)\right)}^{Ή}\right)}^{\frac{1}{Ή}}},e^{-{\left({\xi_{1}\left(-\ln\left({ᵰ}_{{Ґ}_{1}}^{u}\right)\right)}^{Ή}\right)}^{\frac{1}{Ή}}}\right]$. *Then*$\ln\left(1-r^{q}\right)=\left[-{\left({\xi_{1}\left(-\ln\left(1-{\left({ᵯ}_{{Ґ}_{1}}^{l}\right)}^{q}\right)\right)}^{Ή}+{\xi_{2}\left(-\ln\left(1-{\left({ᵯ}_{{Ґ}_{2}}^{l}\right)}^{q}\right)\right)}^{Ή}\right)}^{\frac{1}{Ή}},-{\left({\xi_{1}\left(-\ln\left(1-{\left({ᵯ}_{{Ґ}_{1}}^{u}\right)}^{q}\right)\right)}^{Ή}+{\xi_{2}\left(-\ln\left(1-{\left({ᵯ}_{{Ґ}_{2}}^{u}\right)}^{q}\right)\right)}^{Ή}\right)}^{\frac{1}{Ή}}\right]$. *Through applying the above*, *we have*

$$\text{IV}-q-\text{ROFAAPWA}\left(\Gamma_{1},\Gamma_{2}\right)={\varpi_{1}Ґ}_{1}⨁\varpi_{2}{Ґ}_{2}$$

$$=\left\langle \begin{array}{c} \left(\left[\sqrt[{q}]{1-e^{-{\left({\xi_{1}\left(-\ln\left(1-{ᵯ}_{{Ґ}_{1}}^{l}\right)\right)}^{Ή}\right)}^{\frac{1}{Ή}}}},\sqrt[{q}]{1-e^{-{\left({\xi_{1}\left(-\ln\left(1-{ᵯ}_{{Ґ}_{1}}^{u}\right)\right)}^{Ή}\right)}^{\frac{1}{Ή}}}}\right],\left[e^{-{\left({\xi_{1}\left(-\ln\left({ᵰ}_{{Ґ}_{1}}^{l}\right)\right)}^{Ή}\right)}^{\frac{1}{Ή}}},e^{-{\left({\xi_{1}\left(-\ln\left({ᵰ}_{{Ґ}_{1}}^{u}\right)\right)}^{Ή}\right)}^{\frac{1}{Ή}}}\right]\right)\\ ⨁\left(\left[\sqrt[{q}]{1-e^{-{\left({\xi_{1}\left(-\ln\left(1-{ᵯ}_{{Ґ}_{2}}^{l}\right)\right)}^{Ή}\right)}^{\frac{1}{Ή}}}},\sqrt[{q}]{1-e^{-{\left({\xi_{1}\left(-\ln\left(1-{ᵯ}_{{Ґ}_{2}}^{u}\right)\right)}^{Ή}\right)}^{\frac{1}{Ή}}}}\right],\left[e^{-{\left({\xi_{1}\left(-\ln\left({ᵰ}_{{Ґ}_{2}}^{l}\right)\right)}^{Ή}\right)}^{\frac{1}{Ή}}},e^{-{\left({\xi_{1}\left(-\ln\left({ᵰ}_{{Ґ}_{2}}^{u}\right)\right)}^{Ή}\right)}^{\frac{1}{Ή}}}\right]\right) \end{array}\right\rangle$$

$$=\left\langle \begin{array}{c} \left[\left(\sqrt[{q}]{1-e^{-{\left({\xi_{1}\left(-\ln\left(1-{\left({ᵯ}_{{Ґ}_{1}}^{l}\right)}^{q}\right)\right)}^{Ή}+{\xi_{2}\left(-\ln\left(1-{\left({ᵯ}_{{Ґ}_{2}}^{l}\right)}^{q}\right)\right)}^{Ή}\right)}^{\frac{1}{Ή}}}},\sqrt[{q}]{1-e^{-{\left({\xi_{1}\left(-\ln\left(1-{\left({ᵯ}_{{Ґ}_{1}}^{u}\right)}^{q}\right)\right)}^{Ή}+{\xi_{2}\left(-\ln\left(1-{\left({ᵯ}_{{Ґ}_{2}}^{u}\right)}^{q}\right)\right)}^{Ή}\right)}^{\frac{1}{Ή}}}}\right)\right],\\ \left[\left(\;\sqrt[{q}]{1-e^{-{\left({\xi_{1}\left(-\ln\left(1-{\left({ᵰ}_{{Ґ}_{1}}^{l}\right)}^{q}\right)\right)}^{Ή}+{\xi_{2}\left(-\ln\left(1-{\left({ᵰ}_{{Ґ}_{2}}^{l}\right)}^{q}\right)\right)}^{Ή}\right)}^{\frac{1}{Ή}}}},\sqrt[{q}]{1-e^{-{\left({\xi_{1}\left(-\ln\left(1-{\left({ᵰ}_{{Ґ}_{1}}^{u}\right)}^{q}\right)\right)}^{Ή}+{\xi_{2}\left(-\ln\left(1-{\left({ᵰ}_{{Ґ}_{2}}^{u}\right)}^{q}\right)\right)}^{Ή}\right)}^{\frac{1}{Ή}}}}\right)\right] \end{array}\right\rangle$$

$$=\left\langle \left[\sqrt[{q}]{1-e^{-{\left(\sum_{i=1}^{2}{\xi_{i}\left(-\ln\left(1-{\left({ᵯ}_{{Ґ}_{1}}^{l}\right)}^{q}\right)\right)}^{Ή}\right)}^{\frac{1}{Ή}}}},\sqrt[{q}]{1-e^{-{\left(\sum_{i=1}^{2}{\xi_{i}\left(-\ln\left(1-{\left({ᵯ}_{{Ґ}_{2}}^{u}\right)}^{q}\right)\right)}^{Ή}\right)}^{\frac{1}{Ή}}}}\right],\left[e^{-{\left(\sum_{i=1}^{2}{\xi_{2}\left(-\ln\left({ᵰ}_{{Ґ}_{1}}^{l}\right)\right)}^{Ή}\right)}^{\frac{1}{Ή}}},e^{-{\left(\sum_{i=1}^{2}{\xi_{2}\left(-\ln\left({ᵰ}_{{Ґ}_{2}}^{u}\right)\right)}^{Ή}\right)}^{\frac{1}{Ή}}}\right]\right\rangle$$

Hence satisfied that it is accurate for n=2.

So, let the theorem be accurate for n=k, we get

$$\text{IV}-q-\text{ROFAAPWA}\left(\Gamma_{1},\Gamma_{2},\Gamma_{3},\ldots ,\Gamma_{k}\right)=\begin{array}{c} k\;⊕\\ i=1 \end{array}\left({Ґ}_{i}\varpi_{i}\right)=\left[\sqrt[{q}]{1-e^{-{\left(\sum_{i=1}^{2}{\xi_{i}\left(-\ln\left(1-{\left({ᵯ}_{{Ґ}_{i}}^{l}\right)}^{q}\right)\right)}^{Ή}\right)}^{\frac{1}{Ή}}}},\sqrt[{q}]{1-e^{-{\left(\sum_{i=1}^{2}{\xi_{i}\left(-\ln\left(1-{\left({ᵯ}_{{Ґ}_{i}}^{u}\right)}^{q}\right)\right)}^{Ή}\right)}^{\frac{1}{Ή}}}}\right],\left[e^{-{\left(\sum_{i=1}^{2}{\xi_{2}\left(-\ln\left({ᵰ}_{{Ґ}_{i}}^{l}\right)\right)}^{Ή}\right)}^{\frac{1}{Ή}}},e^{-{\left(\sum_{i=1}^{2}{\xi_{2}\left(-\ln\left({ᵰ}_{{Ґ}_{i}}^{u}\right)\right)}^{Ή}\right)}^{\frac{1}{Ή}}}\right]$$

*Now*, *let the theorem be valid for* n=k+1, *we get*

$$\text{IV}-q-\text{ROFAAPWA}\left({Ґ}_{1},{Ґ}_{2},{Ґ}_{3},\ldots ,{Ґ}_{k},{Ґ}_{k+1}\right)=\begin{array}{c} k\;⊕\\ i=1 \end{array}\left({Ґ}_{i}\varpi_{i}\right)⨁\left({Ґ}_{k+1}\varpi_{k+1}\right)=\left\langle \left(\begin{array}{c} \left[\sqrt[{q}]{1-e^{-{\left(\sum_{i=1}^{k}{\xi_{i}\left(-\ln\left(1-{\left({ᵯ}_{{Ґ}_{i}}^{l}\right)}^{q}\right)\right)}^{Ή}\right)}^{\frac{1}{Ή}}}},\sqrt[{q}]{1-e^{-{\left(\sum_{i=1}^{k}{\xi_{i}\left(-\ln\left(1-{\left({ᵯ}_{{Ґ}_{i}}^{u}\right)}^{q}\right)\right)}^{Ή}\right)}^{\frac{1}{Ή}}}}\right],\\ \left[e^{-{\left(\sum_{i=1}^{k}{\xi_{2}\left(-\ln\left({ᵰ}_{{Ґ}_{i}}^{l}\right)\right)}^{Ή}\right)}^{\frac{1}{Ή}}},e^{-{\left(\sum_{i=1}^{k}{\xi_{2}\left(-\ln\left({ᵰ}_{{Ґ}_{i}}^{u}\right)\right)}^{Ή}\right)}^{\frac{1}{Ή}}}\right] \end{array}\right)⊕\left(\begin{array}{c} \left[\sqrt[{q}]{1-e^{-{\left({\xi_{1}\left(-\ln\left(1-{\left({ᵯ}_{{Ґ}_{k+1}}^{l}\right)}^{q}\right)\right)}^{Ή}\right)}^{\frac{1}{Ή}}}},\sqrt[{q}]{1-e^{-{\left({\xi_{k+1}\left(-\ln\left(1-{\left({ᵯ}_{{Ґ}_{k+1}}^{u}\right)}^{q}\right)\right)}^{Ή}\right)}^{\frac{1}{Ή}}}}\right],\\ \left[e^{-{\left({\xi_{k+1}\left(-\ln\left({ᵰ}_{{Ґ}_{k+1}}^{l}\right)\right)}^{Ή}\right)}^{\frac{1}{Ή}}},e^{-{\left({\xi_{k+1}\left(-\ln\left({ᵰ}_{{Ґ}_{k+1}}^{u}\right)\right)}^{Ή}\right)}^{\frac{1}{Ή}}}\right] \end{array}\right)\right\rangle$$

$$=\left\langle \left(\begin{array}{c} \left[\sqrt[{q}]{1-e^{-{\left({\xi_{1}\left(-\ln\left(1-{\left({ᵯ}_{{Ґ}_{k+1}}^{l}\right)}^{q}\right)\right)}^{Ή}\right)}^{\frac{1}{Ή}}}},\sqrt[{q}]{1-e^{-{\left({\xi_{k+1}\left(-\ln\left(1-{\left({ᵯ}_{{Ґ}_{k+1}}^{u}\right)}^{q}\right)\right)}^{Ή}\right)}^{\frac{1}{Ή}}}}\right],\\ \left[e^{-{\left({\xi_{k+1}\left(-\ln\left({ᵰ}_{{Ґ}_{k+1}}^{l}\right)\right)}^{Ή}\right)}^{\frac{1}{Ή}}},e^{-{\left({\xi_{k+1}\left(-\ln\left({ᵰ}_{{Ґ}_{k+1}}^{u}\right)\right)}^{Ή}\right)}^{\frac{1}{Ή}}}\right] \end{array}\right)\right\rangle$$

*Thus*, *this theorem is valid for* n=k+1. *So*, *it is valid for all real values*.

Theorem 2 (*Idempotency*) *For equal IV-q-ROFVs* ${Ґ}_{i}=\left(\left[{\left({ᵯ}_{i}^{l}\right)}^{q},{\left({ᵯ}_{i}^{u}\right)}^{q}\right],\left[{\left({ᵰ}_{i}^{l}\right)}^{q},{\left({ᵰ}_{i}^{u}\right)}^{q}\right]\right)$, *that is* ${Ґ}_{i}=Ґ$, *then*

$$\text{IV}-q-\text{ROFPAAWA}\left({Ґ}_{1},{Ґ}_{2},{Ґ}_{3},\ldots ,{Ґ}_{n}\right)=Ґ\text{.}$$

Proof: Since $Ґ=\left(\left[{\left({ᵯ}^{l}\right)}^{q},{\left({ᵯ}^{u}\right)}^{q}\right],\left[{\left({ᵰ}^{l}\right)}^{q},{\left({ᵰ}^{u}\right)}^{q}\right]\right)$ then by Eq. (9):

$$\text{IV}‐q‐\text{ROFAAPWA}\left({Ґ}_{1},{Ґ}_{2},{Ґ}_{3},\ldots ,{Ґ}_{n}\right)=\begin{array}{c} n\;⊕\\ \varpi =1 \end{array}\left({Ґ}_{i}\varpi_{i}\right)=\left\langle \left(\begin{array}{c} \left[\sqrt[{q}]{1‐e^{‐{\left(\sum_{i=1}^{n}{\xi_{i}\left(‐\ln\left(1‐{\left({ᵯ}_{{Ґ}_{i}}^{l}\right)}^{q}\right)\right)}^{Ή}\right)}^{\frac{1}{Ή}}}},\sqrt[{q}]{1‐e^{‐{\left(\sum_{i=1}^{n}{\xi_{i}\left(‐\ln\left(1‐{\left({ᵯ}_{{Ґ}_{i}}^{u}\right)}^{q}\right)\right)}^{Ή}\right)}^{\frac{1}{Ή}}}}\right],\\ \left[e^{‐{\left(\sum_{i=1}^{n}{\xi_{i}\left(‐\ln\left({ᵰ}_{{Ґ}_{i}}^{l}\right)\right)}^{Ή}\right)}^{\frac{1}{Ή}}},e^{‐{\left(\sum_{i=1}^{n}{\xi_{i}\left(‐\ln\left({ᵰ}_{{Ґ}_{i}}^{u}\right)\right)}^{Ή}\right)}^{\frac{1}{Ή}}}\right] \end{array}\right)\right\rangle =\left\langle \left[\sqrt[{q}]{1‐e^{‐{\left({\xi_{i}\left(‐\ln\left(1‐{\left({ᵯ}_{{Ґ}_{i}}^{l}\right)}^{q}\right)\right)}^{Ή}\right)}^{\frac{1}{Ή}}}},\sqrt[{q}]{1‐e^{‐{\left({\xi_{i}\left(‐\ln\left(1‐{\left({ᵯ}_{{Ґ}_{i}}^{u}\right)}^{q}\right)\right)}^{Ή}\right)}^{\frac{1}{Ή}}}}\right]\;\left[e^{‐{\left({\xi_{i}\left(‐\ln\left({ᵰ}_{{Ґ}_{i}}^{l}\right)\right)}^{Ή}\right)}^{\frac{1}{Ή}}},e^{‐{\left({\xi_{i}\left(‐\ln\left({ᵰ}_{{Ґ}_{i}}^{u}\right)\right)}^{Ή}\right)}^{\frac{1}{Ή}}}\right]\right\rangle$$

$$=\left\langle \left[\sqrt[{q}]{1-e^{\ln\left(1-{\left({ᵯ}_{{Ґ}_{i}}^{l}\right)}^{q}\right)}},\sqrt[{q}]{1-e^{\ln\left(1-{\left({ᵯ}_{{Ґ}_{i}}^{u}\right)}^{q}\right)}}\right],\left[e^{\ln\left({ᵰ}_{{Ґ}_{i}}^{l}\right)},e^{\ln\left({ᵰ}_{{Ґ}_{i}}^{u}\right)}\right]\right\rangle$$

$$=\left\langle \left[\sqrt[{q}]{{\left({ᵯ}_{{Ґ}_{i}}^{l}\right)}^{q}},\sqrt[{q}]{{\left({ᵯ}_{{Ґ}_{i}}^{u}\right)}^{q}}\right],\left[{ᵰ}_{{Ґ}_{i}}^{l},{ᵰ}_{{Ґ}_{i}}^{u}\right]\right\rangle =\left(\left[{ᵯ}_{{Ґ}_{i}}^{l},{ᵯ}_{{Ґ}_{i}}^{u}\right],\left[{ᵰ}_{{Ґ}_{i}}^{l},{ᵰ}_{{Ґ}_{i}}^{u}\right]\right)=Ґ$$

*Thus*, $\text{IV}-q-\text{ROFPAAWA}\left({Ґ}_{1},{Ґ}_{2},{Ґ}_{3},\ldots ,{Ґ}_{n}\right)=Ґ$ *holds*.

Theorem 3 (Boundedness) If ${Ґ}_{i}=\left(\left[{\left({ᵯ}_{i}^{l}\right)}^{q},{\left({ᵯ}_{i}^{u}\right)}^{q}\right],\left[{\left({ᵰ}_{i}^{l}\right)}^{q},{\left({ᵰ}_{i}^{u}\right)}^{q}\right]\right)$ be many equal IV-q-ROFVs, that is ${Ґ}^{-}=\min\left({Ґ}_{1},{Ґ}_{2},{Ґ}_{3},\ldots ,{Ґ}_{n}\right)$ and ${Ґ}^{+}=\max\left({Ґ}_{1},{Ґ}_{2},{Ґ}_{3},\ldots ,{Ґ}_{n}\right)$ then ${Ґ}^{-}\leq \text{IV}-q-\text{ROFAAPWA}\left({Ґ}_{1},{Ґ}_{2},{Ґ}_{3},\ldots ,{Ґ}_{n}\right)\leq {Ґ}^{+}$

Proof: For IV-q-ROFVs ${Ґ}_{i}=\left(\left[{\left({ᵯ}_{i}^{l}\right)}^{q},{\left({ᵯ}_{i}^{u}\right)}^{q}\right],\left[{\left({ᵰ}_{i}^{l}\right)}^{q},{\left({ᵰ}_{i}^{u}\right)}^{q}\right]\right)\;\left(i=1,2,\ldots ,n\right)$, Consider ${Ґ}^{-}=\min\left({Ґ}_{1},{Ґ}_{2},{Ґ}_{3},\ldots ,{Ґ}_{n}\right)=\left(\left[{ᵯ}_{i}^{-l},{ᵯ}_{i}^{-u}\right],\left[{ᵰ}_{i}^{-l},{ᵰ}_{i}^{-u}\right]\right)$ and ${Ґ}^{+}=\max\left({Ґ}_{1},{Ґ}_{2},{Ґ}_{3},\ldots ,{Ґ}_{n}\right)=\left(\left[{ᵯ}_{i}^{+l},{ᵯ}_{i}^{+u}\right],\left[{ᵰ}_{i}^{+l},{ᵰ}_{i}^{+u}\right]\right)$. We have ${Ґ}^{-}=\min\left(\left[{ᵯ}_{{Ґ}_{i}}^{l},{ᵯ}_{{Ґ}_{i}}^{u}\right]\right),{Ґ}^{-}=\max\left(\left[{ᵰ}_{{Ґ}_{i}}^{l},{ᵰ}_{{Ґ}_{i}}^{u}\right]\right),\Gamma^{+}=\max\left(\left[{ᵯ}_{{Ґ}_{i}}^{l},{ᵯ}_{{Ґ}_{i}}^{u}\right]\right),\Gamma^{+}=\min\left(\left[{ᵰ}_{{Ґ}_{i}}^{l},{ᵰ}_{{Ґ}_{i}}^{u}\right]\right)$.

$$\left[\sqrt[{q}]{1-e^{-{\left(\sum_{i=1}^{n}{\xi_{i}\left(-\ln\left(1-{\left({ᵯ}_{{Ґ}_{i}}^{-l}\right)}^{q}\right)\right)}^{Ή}\right)}^{\frac{1}{Ή}}}},\sqrt[{q}]{1-e^{-{\left(\sum_{i=1}^{n}{\xi_{i}\left(-\ln\left(1-{\left({ᵯ}_{{Ґ}_{i}}^{-u}\right)}^{q}\right)\right)}^{Ή}\right)}^{\frac{1}{Ή}}}}\right]\leq \left[\sqrt[{q}]{1-e^{-{\left(\sum_{i=1}^{n}{\xi_{i}\left(-\ln\left(1-{\left({ᵯ}_{{Ґ}_{i}}^{l}\right)}^{q}\right)\right)}^{Ή}\right)}^{\frac{1}{Ή}}}},\sqrt[{q}]{1-e^{-{\left(\sum_{i=1}^{n}{\xi_{i}\left(-\ln\left(1-{\left({ᵯ}_{{Ґ}_{i}}^{u}\right)}^{q}\right)\right)}^{Ή}\right)}^{\frac{1}{Ή}}}}\right]\leq \left[\sqrt[{q}]{1-e^{-{\left(\sum_{i=1}^{n}{\xi_{i}\left(-\ln\left(1-{\left({ᵯ}_{{Ґ}_{i}}^{+l}\right)}^{q}\right)\right)}^{Ή}\right)}^{\frac{1}{Ή}}}},\sqrt[{q}]{1-e^{-{\left(\sum_{i=1}^{n}{\xi_{i}\left(-\ln\left(1-{\left({ᵯ}_{{Ґ}_{i}}^{+u}\right)}^{q}\right)\right)}^{Ή}\right)}^{\frac{1}{Ή}}}}\right]$$

$$\left[e^{-{\left(\sum_{i=1}^{n}{\xi_{i}\left(-\ln\left({ᵰ}_{{Ґ}_{i}}^{-l}\right)\right)}^{Ή}\right)}^{\frac{1}{Ή}}},e^{-{\left(\sum_{i=1}^{n}{\xi_{i}\left(-\ln\left({ᵰ}_{{Ґ}_{i}}^{-u}\right)\right)}^{Ή}\right)}^{\frac{1}{Ή}}}\right]\leq \left[e^{-{\left(\sum_{i=1}^{n}{\xi_{i}\left(-\ln\left({ᵰ}_{{Ґ}_{i}}^{l}\right)\right)}^{Ή}\right)}^{\frac{1}{Ή}}},e^{-{\left(\sum_{i=1}^{n}{\xi_{i}\left(-\ln\left({ᵰ}_{{Ґ}_{i}}^{u}\right)\right)}^{Ή}\right)}^{\frac{1}{Ή}}}\right]\leq \left[e^{-{\left(\sum_{i=1}^{n}{\xi_{i}\left(-\ln\left({ᵰ}_{{Ґ}_{i}}^{+l}\right)\right)}^{Ή}\right)}^{\frac{1}{Ή}}},e^{-{\left(\sum_{i=1}^{n}{\xi_{i}\left(-\ln\left({ᵰ}_{{Ґ}_{i}}^{+u}\right)\right)}^{Ή}\right)}^{\frac{1}{Ή}}}\right]$$

Therefore, ${Ґ}^{-}\leq \text{IV}-q-\text{ROFAAPWA}\left({Ґ}_{1},{Ґ}_{2},{Ґ}_{3},\ldots ,{Ґ}_{n}\right)\leq {Ґ}^{+}$.

Theorem 4 (*Monotonicity*) *For two sets of IV-q-ROFVs* ${Ґ}_{i}=\left(\left[{\left({ᵯ}_{i}^{l}\right)}^{q},{\left({ᵯ}_{i}^{u}\right)}^{q}\right],\left[{\left({ᵰ}_{i}^{l}\right)}^{q},{\left({ᵰ}_{i}^{u}\right)}^{q}\right]\right)\;\left(i=1,2,\ldots ,n\right)$, *if* ${Ґ}_{i}\leq {Ґ}_{i}^{\prime }$, *then*

$$\text{IV}-q-\text{ROFAAPWA}\left({Ґ}_{1},{Ґ}_{2},{Ґ}_{3},\ldots ,{Ґ}_{n}\right)\leq \text{IV}-q-\text{ROFAAPWA}\left({Ґ}_{1}^{\prime },{Ґ}_{2}^{\prime },{Ґ}_{3}^{\prime },\ldots ,{Ґ}_{n}^{\prime }\right)$$

Theorem 5 *For a collection of IV-q-ROFVs*${Ґ}_{i}=\left(\left[{\left({ᵯ}_{i}^{l}\right)}^{q},{\left({ᵯ}_{i}^{u}\right)}^{q}\right],\left[{\left({ᵰ}_{i}^{l}\right)}^{q},{\left({ᵰ}_{i}^{u}\right)}^{q}\right]\right)$*in this*i=1,2,3,…,n, *the calculated outcome is a q-ROFN through*Definition 1, *also an IV-q-ROFV*. *The AO can be shown in* Equation (10). *as follows*:

(10) $$\text{IV}-q-\text{ROFAAPWG}\left({Ґ}_{1},{Ґ}_{2},{Ґ}_{3},\ldots ,{Ґ}_{n}\right)=\begin{array}{c} n\\ ⨂\\ \varpi =1 \end{array}\left({Ґ}_{i}^{\varpi_{i}}\right)=\begin{array}{c} \left\langle \begin{array}{c} \left[\sqrt[{q}]{e^{-{\left(\sum_{i=1}^{n}{\xi_{i}\left(-\ln\;{\left({ᵯ}_{{Ґ}_{i}}^{l}\right)}^{q}\right)}^{Ή}\right)}^{\frac{1}{Ή}}}},\sqrt[{q}]{e^{-{\left(\sum_{i=1}^{k+1}{\xi_{i}\left(-\ln\;{\left({ᵯ}_{{Ґ}_{i}}^{u}\right)}^{q}\right)}^{Ή}\right)}^{\frac{1}{Ή}}}}\right],\\ \left[1-e^{-{\left(\sum_{i=1}^{n}{\xi_{i}\left(-\ln\left(1-{ᵰ}_{{Ґ}_{i}}^{l}\right)\right)}^{Ή}\right)}^{\frac{1}{Ή}}},1-e^{-{\left(\sum_{i=1}^{n}{\xi_{i}\left(-\ln\left(1-\left({ᵰ}_{{Ґ}_{i}}^{u}\right)\right)\right)}^{Ή}\right)}^{\frac{1}{Ή}}}\right] \end{array}\right\rangle \end{array}$$

*In this* $\xi_{i}$ *and* i=1,2,…,n *be the collection of assimilated weights such as*:

$$\xi_{i}=\frac{\varpi_{i}\left(1+A\left(\beta_{i}\right)\right)}{\sum_{i=1}^{n}\varpi_{i}\left(1+A\left(\beta_{i}\right)\right)}$$

So, $\xi_{i}>0,\sum_{i=1}^{n}\xi_{i}=1$.

Proof: Using the AA operation, we apply a mathematical induction technique to satisfy that the theorem is valid for n=2.

$$\varpi_{1}{Ґ}_{1}=\left\langle \left[\sqrt[{q}]{e^{-{\left({\xi_{1}\left(-\ln\;{\left({ᵯ}_{{Ґ}_{1}}^{l}\right)}^{q}\right)}^{Ή}\right)}^{\frac{1}{Ή}}}},\sqrt[{q}]{e^{-{\left({\xi_{1}\left(-\ln\;{\left({ᵯ}_{{Ґ}_{1}}^{u}\right)}^{q}\right)}^{Ή}\right)}^{\frac{1}{Ή}}}}\right],\left[1-e^{-{\left({\xi_{1}\left(-\ln\left(1-{ᵰ}_{{Ґ}_{1}}^{l}\right)\right)}^{Ή}\right)}^{\frac{1}{Ή}}},1-e^{-{\left({\xi_{1}\left(-\ln\left(1-{ᵰ}_{{Ґ}_{1}}^{u}\right)\right)}^{Ή}\right)}^{\frac{1}{Ή}}}\right]\right\rangle$$

$$\varpi_{2}{Ґ}_{2}=\left\langle \left[\sqrt[{q}]{e^{-{\left({\xi_{1}\left(-\ln\;{\left({ᵯ}_{{Ґ}_{2}}^{l}\right)}^{q}\right)}^{Ή}\right)}^{\frac{1}{Ή}}}},\sqrt[{q}]{e^{-{\left({\xi_{1}\left(-\ln\;{\left({ᵯ}_{{Ґ}_{2}}^{u}\right)}^{q}\right)}^{Ή}\right)}^{\frac{1}{Ή}}}}\right],\left[1-e^{-{\left({\xi_{1}\left(-\ln\left(1-{ᵰ}_{{Ґ}_{2}}^{l}\right)\right)}^{Ή}\right)}^{\frac{1}{Ή}}},1-e^{-{\left({\xi_{1}\left(-\ln\left(1-{ᵰ}_{{Ґ}_{2}}^{u}\right)\right)}^{Ή}\right)}^{\frac{1}{Ή}}}\right]\right\rangle$$

*Now*, *by using*Definition 1, *we obtained*

$$\text{IV}-q-\text{ROFAAPWG}\left({Ґ}_{1},{Ґ}_{2}\right)={Ґ}_{1}^{\varpi_{1}}⨂{Ґ}_{2}^{\varpi_{2}}=$$

$$\left\langle \begin{array}{c} \left\langle \left[\sqrt[{q}]{e^{-{\left({\xi_{1}\left(-\ln\;{\left({ᵯ}_{{Ґ}_{1}}^{l}\right)}^{q}\right)}^{Ή}\right)}^{\frac{1}{Ή}}}},\sqrt[{q}]{e^{-{\left({\xi_{1}\left(-\ln\;{\left({ᵯ}_{{Ґ}_{1}}^{u}\right)}^{q}\right)}^{Ή}\right)}^{\frac{1}{Ή}}}}\right],\left[1-e^{-{\left({\xi_{1}\left(-\ln\left(1-{ᵰ}_{{Ґ}_{1}}^{l}\right)\right)}^{Ή}\right)}^{\frac{1}{Ή}}},1-e^{-{\left({\xi_{1}\left(-\ln\left(1-{ᵰ}_{{Ґ}_{1}}^{u}\right)\right)}^{Ή}\right)}^{\frac{1}{Ή}}}\right]\right\rangle \\ ⨂\left\langle \left[\sqrt[{q}]{e^{-{\left({\xi_{1}\left(-\ln\;{\left({ᵯ}_{{Ґ}_{2}}^{l}\right)}^{q}\right)}^{Ή}\right)}^{\frac{1}{Ή}}}},\sqrt[{q}]{e^{-{\left({\xi_{1}\left(-\ln\;{\left({ᵯ}_{{Ґ}_{2}}^{u}\right)}^{q}\right)}^{Ή}\right)}^{\frac{1}{Ή}}}}\right],\left[1-e^{-{\left({\xi_{1}\left(-\ln\left(1-{ᵰ}_{{Ґ}_{2}}^{l}\right)\right)}^{Ή}\right)}^{\frac{1}{Ή}}},1-e^{-{\left({\xi_{1}\left(-\ln\left(1-{ᵰ}_{{Ґ}_{2}}^{u}\right)\right)}^{Ή}\right)}^{\frac{1}{Ή}}}\right]\right\rangle \end{array}\right\rangle$$

$$=\left\langle \begin{array}{c} \left[\sqrt[{q}]{e^{-{\left({\xi_{1}\left(-\ln\;{\left({ᵯ}_{{Ґ}_{1}}^{l}\right)}^{q}\right)}^{Ή}+{\xi_{2}\left(-\ln\;{\left({ᵯ}_{{Ґ}_{2}}^{l}\right)}^{q}\right)}^{Ή}\right)}^{\frac{1}{Ή}}}},\sqrt[{q}]{e^{-{\left({\xi_{1}\left(-\ln\;{\left({ᵯ}_{{Ґ}_{1}}^{u}\right)}^{q}\right)}^{Ή}+{\xi_{2}\left(-\ln\;{\left({ᵯ}_{{Ґ}_{2}}^{u}\right)}^{q}\right)}^{Ή}\right)}^{\frac{1}{Ή}}}}\right],\\ \left[1-e^{-{\left({\xi_{1}\left(-\ln\left(1-{ᵰ}_{{Ґ}_{1}}^{l}\right)\right)}^{Ή}+{\xi_{2}\left(-\ln\left(1-{ᵰ}_{{Ґ}_{2}}^{l}\right)\right)}^{Ή}\right)}^{\frac{1}{Ή}}},1-e^{-{\left({\xi_{1}\left(-\ln\left(1-{ᵰ}_{{Ґ}_{1}}^{u}\right)\right)}^{Ή}+{\xi_{2}\left(-\ln\left(1-{ᵰ}_{{Ґ}_{2}}^{u}\right)\right)}^{Ή}\right)}^{\frac{1}{Ή}}},\right] \end{array}\right\rangle$$

$$=\left\langle \left[\sqrt[{q}]{e^{-{\left(\sum_{i=1}^{2}{\xi_{i}\left(-\ln\;{\left({ᵯ}_{{Ґ}_{1}}^{l}\right)}^{q}\right)}^{Ή}\right)}^{\frac{1}{Ή}}}},\sqrt[{q}]{e^{-{\left(\sum_{i=1}^{2}{\xi_{i}\left(-\ln\;{\left({ᵯ}_{{Ґ}_{1}}^{u}\right)}^{q}\right)}^{Ή}\right)}^{\frac{1}{Ή}}}}\right],\left[1-e^{-{\left(\sum_{i=1}^{2}{\xi_{2}\left(-\ln\left(1-{ᵰ}_{{Ґ}_{2}}^{u}\right)\right)}^{Ή}\right)}^{\frac{1}{Ή}}},1-e^{-{\left(\sum_{i=1}^{2}{\xi_{2}\left(-\ln\left(1-{ᵰ}_{{Ґ}_{2}}^{u}\right)\right)}^{Ή}\right)}^{\frac{1}{Ή}}}\right]\right\rangle$$

Hence, the theorem is valid for n=2.

So, let this theorem be accurate for n=k. We get

$$\text{IV}-q-\text{ROFAAPWG}\left({Ґ}_{1},{Ґ}_{2},{Ґ}_{3},\ldots ,{Ґ}_{k}\right)=\begin{array}{c} k\\ ⨂\\ i=1 \end{array}\left({Ґ}_{i}^{\varpi_{i}}\right)=\left\langle \begin{array}{c} \left[\sqrt[{q}]{e^{-{\left(\sum_{i=1}^{2}{\xi_{i}\left(-\ln\;{\left({ᵯ}_{{Ґ}_{i}}^{l}\right)}^{q}\right)}^{Ή}\right)}^{\frac{1}{Ή}}}},\sqrt[{q}]{e^{-{\left(\sum_{i=1}^{2}{\xi_{i}\left(-\ln\;{\left({ᵯ}_{{Ґ}_{i}}^{u}\right)}^{q}\right)}^{Ή}\right)}^{\frac{1}{Ή}}}}\right],\\ \left[1-e^{-{\left(\sum_{i=1}^{k}{\xi_{i}\left(-\ln\left(1-{ᵰ}_{{Ґ}_{i}}^{l}\right)\right)}^{Ή}\right)}^{\frac{1}{Ή}}},1-e^{-{\left(\sum_{i=1}^{k}{\xi_{i}\left(-\ln\left(1-{ᵰ}_{{Ґ}_{i}}^{u}\right)\right)}^{Ή}\right)}^{\frac{1}{Ή}}}\right] \end{array}\right\rangle$$

*Now*, *let this theorem be valid for* n=k+1. *We get*

**Figure:**
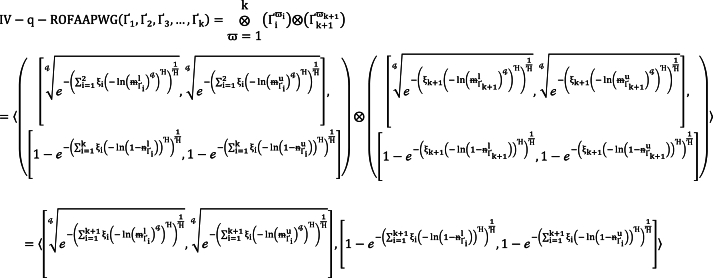

Hence, this theorem is valid for n=k+1. So, this statement is valid for all real values lying between [0,1].

Theorem 6 (*Idempotency*) *For many equal IV-q-ROFVs* ${Ґ}_{i}=\left(\left[{\left({ᵯ}_{i}^{l}\right)}^{q},{\left({ᵯ}_{i}^{u}\right)}^{q}\right],\left[{\left({ᵰ}_{i}^{l}\right)}^{q},{\left({ᵰ}_{i}^{u}\right)}^{q}\right]\right)$, *that is* ${Ґ}_{i}=Ґ$, *the*

$$\text{IV}-q-\text{ROFAAPWG}\left({Ґ}_{1},{Ґ}_{2},{Ґ}_{3},\ldots ,{Ґ}_{k}\right)=Ґ\text{.}$$

Proof: Since ${Ґ}_{i}=\left(\left[{\left({ᵯ}_{i}^{l}\right)}^{q},{\left({ᵯ}_{i}^{u}\right)}^{q}\right],\left[{\left({ᵰ}_{i}^{l}\right)}^{q},{\left({ᵰ}_{i}^{u}\right)}^{q}\right]\right)\;\left(i=1,2,\ldots ,n\right)$ through Equation (9).

$$\text{IV}-q-\text{ROFAAPWG}\left({Ґ}_{1},{Ґ}_{2},{Ґ}_{3},\ldots ,{Ґ}_{k}\right)=\begin{array}{c} k\\ ⨂\\ i=1 \end{array}\left({Ґ}_{i}^{\varpi_{i}}\right)=\left\langle \left(\begin{array}{c} \left[\sqrt[{q}]{e^{-{\left(\sum_{i=1}^{n}{\xi_{i}\left(-\ln\;{\left({ᵯ}_{{Ґ}_{i}}^{l}\right)}^{q}\right)}^{Ή}\right)}^{\frac{1}{Ή}}}},\sqrt[{q}]{e^{-{\left(\sum_{i=1}^{n}{\xi_{i}\left(-\ln\;{\left({ᵯ}_{{Ґ}_{i}}^{u}\right)}^{q}\right)}^{Ή}\right)}^{\frac{1}{Ή}}}}\right],\\ \left[1-e^{-{\left(\sum_{i=1}^{n}{\xi_{i}\left(-\ln\left(1-{ᵰ}_{{Ґ}_{i}}^{l}\right)\right)}^{Ή}\right)}^{\frac{1}{Ή}}},1-e^{-{\left(\sum_{i=1}^{n}{\xi_{i}\left(-\ln\left(1-{ᵰ}_{{Ґ}_{i}}^{u}\right)\right)}^{Ή}\right)}^{\frac{1}{Ή}}}\right] \end{array}\right)\right\rangle$$

$$=\left\langle \left[\sqrt[{q}]{e^{-{\left({\xi_{i}\left(-\ln\;{\left({ᵯ}_{{Ґ}_{i}}^{l}\right)}^{q}\right)}^{Ή}\right)}^{\frac{1}{Ή}}}},\sqrt[{q}]{e^{-{\left({\xi_{i}\left(-\ln\;{\left({ᵯ}_{{Ґ}_{i}}^{u}\right)}^{q}\right)}^{Ή}\right)}^{\frac{1}{Ή}}}}\right],\left[1-e^{-{\left({\xi_{i}\left(-\ln\left(1-{ᵰ}_{{Ґ}_{i}}^{l}\right)\right)}^{Ή}\right)}^{\frac{1}{Ή}}},1-e^{-{\left({\xi_{i}\left(-\ln\left(1-{ᵰ}_{{Ґ}_{i}}^{u}\right)\right)}^{Ή}\right)}^{\frac{1}{Ή}}}\right]\right\rangle$$

$$=\left\langle \left[\sqrt[{q}]{e^{\ln\;{\left({ᵯ}_{{Ґ}_{i}}^{l}\right)}^{q}}},\sqrt[{q}]{e^{\ln\;{\left({ᵯ}_{{Ґ}_{i}}^{u}\right)}^{q}}}\right],\left[1-e^{\ln\left(1-{ᵰ}_{{Ґ}_{i}}^{l}\right)},1-e^{\ln\left(1-{ᵰ}_{{Ґ}_{i}}^{u}\right)}\right]\right\rangle$$

$$=\left\langle \left[\sqrt[{q}]{{\left({ᵯ}_{{Ґ}_{i}}^{l}\right)}^{q}},\sqrt[{q}]{{\left({ᵯ}_{{Ґ}_{i}}^{l}\right)}^{q}}\right],\left[{ᵰ}_{{Ґ}_{i}}^{l},{ᵰ}_{{Ґ}_{i}}^{l}\right]\right\rangle =\left(\left[{ᵯ}_{{Ґ}_{i}}^{l},{ᵯ}_{{Ґ}_{i}}^{u}\right],\left[{ᵰ}_{{Ґ}_{i}}^{l},{ᵰ}_{{Ґ}_{i}}^{u}\right]\right)=Ґ$$

*Thus*, $\text{IV}-q-\text{ROFAAPWG}\left({Ґ}_{1},{Ґ}_{2},{Ґ}_{3},\ldots ,{Ґ}_{k}\right)=Ґ\text{.}$ *holds*.

Theorem 7 (Boundedness) For many equal IV-q-ROFVs ${Ґ}_{i}=\left(\left[{\left({ᵯ}_{i}^{l}\right)}^{q},{\left({ᵯ}_{i}^{u}\right)}^{q}\right],\left[{\left({ᵰ}_{i}^{l}\right)}^{q},{\left({ᵰ}_{i}^{u}\right)}^{q}\right]\right)$, that is ${Ґ}^{-}=\min\left({Ґ}_{1},{Ґ}_{2},{Ґ}_{3},\ldots ,{Ґ}_{n}\right)$ and ${Ґ}^{+}=\max\left({Ґ}_{1},{Ґ}_{2},{Ґ}_{3},\ldots ,{Ґ}_{n}\right)$ the ${Ґ}^{-}\leq \text{IV}-q-\text{ROFAAPWG}\left({Ґ}_{1},{Ґ}_{2},{Ґ}_{3},\ldots ,{Ґ}_{n}\right)\leq {Ґ}^{+}$

Proof**:** For IV-q-ROFVs ${Ґ}_{i}=\left(\left[{\left({ᵯ}_{i}^{l}\right)}^{q},{\left({ᵯ}_{i}^{u}\right)}^{q}\right],\left[{\left({ᵰ}_{i}^{l}\right)}^{q},{\left({ᵰ}_{i}^{u}\right)}^{q}\right]\right)\text{,}$ consider ${Ґ}^{-}=\min\left({Ґ}_{1},{Ґ}_{2},{Ґ}_{3},\ldots ,{Ґ}_{n}\right)=\left(\left[{ᵯ}_{i}^{-l},{ᵯ}_{i}^{-u}\right],\left[{ᵰ}_{i}^{-l},{ᵰ}_{i}^{-u}\right]\right)$ and ${Ґ}^{+}=\max\left({Ґ}_{1},{Ґ}_{2},{Ґ}_{3},\ldots ,{Ґ}_{n}\right)=\left(\left[{ᵯ}_{i}^{+l},{ᵯ}_{i}^{+u}\right],\left[{ᵰ}_{i}^{+l},{ᵰ}_{i}^{+u}\right]\right)$. We have ${Ґ}^{-}=\min\left(\left[{ᵯ}_{{Ґ}_{i}}^{l},{ᵯ}_{{Ґ}_{i}}^{u}\right]\right),{Ґ}^{-}=\max\left(\left[{ᵰ}_{{Ґ}_{i}}^{l},{ᵰ}_{{Ґ}_{i}}^{u}\right]\right),\Gamma^{+}=\max\left(\left[{ᵯ}_{{Ґ}_{i}}^{l},{ᵯ}_{{Ґ}_{i}}^{u}\right]\right),\Gamma^{+}=\min\left(\left[{ᵰ}_{{Ґ}_{i}}^{l},{ᵰ}_{{Ґ}_{i}}^{u}\right]\right)$.

$$\left[\sqrt[{q}]{e^{-{\left(\sum_{i=1}^{n}{\xi_{i}\left(-\ln\left({\left({ᵯ}_{{Ґ}_{i}}^{-l}\right)}^{q}\right)\right)}^{Ή}\right)}^{\frac{1}{Ή}}}},\sqrt[{q}]{e^{-{\left(\sum_{i=1}^{n}{\xi_{i}\left(-\ln\left({\left({ᵯ}_{{Ґ}_{i}}^{-u}\right)}^{q}\right)\right)}^{Ή}\right)}^{\frac{1}{Ή}}}}\right]\leq \left[\sqrt[{q}]{e^{-{\left(\sum_{i=1}^{n}{\xi_{i}\left(-\ln\left({\left({ᵯ}_{{Ґ}_{i}}^{l}\right)}^{q}\right)\right)}^{Ή}\right)}^{\frac{1}{Ή}}}},\sqrt[{q}]{e^{-{\left(\sum_{i=1}^{n}{\xi_{i}\left(-\ln\left({\left({ᵯ}_{{Ґ}_{i}}^{u}\right)}^{q}\right)\right)}^{Ή}\right)}^{\frac{1}{Ή}}}}\right]\leq \left[\sqrt[{q}]{e^{-{\left(\sum_{i=1}^{n}{\xi_{i}\left(-\ln\left({\left({ᵯ}_{{Ґ}_{i}}^{+l}\right)}^{q}\right)\right)}^{Ή}\right)}^{\frac{1}{Ή}}}},\sqrt[{q}]{e^{-{\left(\sum_{i=1}^{n}{\xi_{i}\left(-\ln\left({\left({ᵯ}_{{Ґ}_{i}}^{+u}\right)}^{q}\right)\right)}^{Ή}\right)}^{\frac{1}{Ή}}}}\right]$$

$$\left[{1-e}^{-{\left(\sum_{i=1}^{n}{\xi_{i}\left(-\ln\left(1-{ᵰ}_{{Ґ}_{i}}^{-l}\right)\right)}^{Ή}\right)}^{\frac{1}{Ή}}},{1-e}^{-{\left(\sum_{i=1}^{n}{\xi_{i}\left(-\ln\left({1-ᵰ}_{{Ґ}_{i}}^{-u}\right)\right)}^{Ή}\right)}^{\frac{1}{Ή}}}\right]\leq \left[{1-e}^{-{\left(\sum_{i=1}^{n}{\xi_{i}\left(-\ln\left(1-{ᵰ}_{{Ґ}_{i}}^{l}\right)\right)}^{Ή}\right)}^{\frac{1}{Ή}}},{1-e}^{-{\left(\sum_{i=1}^{n}{\xi_{i}\left(-\ln\left({1-ᵰ}_{{Ґ}_{i}}^{u}\right)\right)}^{Ή}\right)}^{\frac{1}{Ή}}}\right]\leq \left[{1-e}^{-{\left(\sum_{i=1}^{n}{\xi_{i}\left(-\ln\left(1-{ᵰ}_{{Ґ}_{i}}^{+l}\right)\right)}^{Ή}\right)}^{\frac{1}{Ή}}},{1-e}^{-{\left(\sum_{i=1}^{n}{\xi_{i}\left(-\ln\left({1-ᵰ}_{{Ґ}_{i}}^{+u}\right)\right)}^{Ή}\right)}^{\frac{1}{Ή}}}\right]$$

Therefore, ${Ґ}^{-}\leq \text{IV}-q-\text{ROFAAPWG}\left({Ґ}_{1},{Ґ}_{2},{Ґ}_{3},\ldots ,{Ґ}_{n}\right)\leq {Ґ}^{+}$.

Theorem 8 (*Monotonicity*) *For two sets of IV-q-ROFVs* ${Ґ}_{i}=\left(\left[{\left({ᵯ}_{i}^{l}\right)}^{q},{\left({ᵯ}_{i}^{u}\right)}^{q}\right],\left[{\left({ᵰ}_{i}^{l}\right)}^{q},{\left({ᵰ}_{i}^{u}\right)}^{q}\right]\right)\;\left(i=1,2,\ldots ,n\right)$, *if* ${Ґ}_{i}\leq {Ґ}_{i}^{\prime }$, *then*

$$\text{IV}-q-\text{ROFAAPWG}\left({Ґ}_{1},{Ґ}_{2},{Ґ}_{3},\ldots ,{Ґ}_{n}\right)\leq \text{IV}-q-\text{ROFAAPWG}\left({Ґ}_{1}^{\prime },{Ґ}_{2}^{\prime },{Ґ}_{3}^{\prime },\ldots ,{Ґ}_{n}^{\prime }\right)$$

## 4 Multi-attribute group decision-making algorithm based on the q-ROFSs

Here in, using the suggested method in an IV-q-RFS environment, we construct a MAGDM methodology. suppose $ɉ=\left\{{ɉ}_{1},{ɉ}_{2},\ldots ,{ɉ}_{ɱ}\right\}$ are ${ᶆ}^{\text{th}}$ attributes and $ȁ=\left\{{ȁ}_{1},{ȁ}_{2},\ldots ,{ȁ}_{ȵ}\right\}$ are said to ɳ alternatives for selection. Also $\varpi_{i}$ be the WV of the attributes that are allotted through the decision-makers, and its all-time fulfils that $\sum_{i=1}^{n}\varpi_{i}=1$. The WV of the decision-maker $D_{p}$ is represented through $\gamma_{p}\;\left(k=1,2,\ldots ,p\right)$ and ${0\leq \gamma }_{i}\geq 1$. Through applying the IV-q-ROFS data, make a decision matrix $Ų={\left({Ŝ}_{k}\right)}_{ɱ\times ȵ}$ in this IV-q-ROFS information ${Ґ}_{i}=\left(\left[{\left({ᶆ}_{i}^{l}\right)}^{q},{\left({ᶆ}_{i}^{u}\right)}^{q}\right],\left[{\left({ᵰ}_{i}^{l}\right)}^{q},{\left({ᵰ}_{i}^{u}\right)}^{q}\right]\right)$ be the collection of attributes $x_{j}$ the $D_{p}$ provide alternatives $e_{j}$. ${\;s}_{k}=\left(\left[{ᶆ}_{k},{ᶆ}_{k}\right],\left[{ᵰ}_{k},{ᵰ}_{k}\right]\right)$ and assessed the values of the $e_{j}$ all the time lie in $0\leq \left[\left({\left({ᶆ}_{k}^{l}\right)}^{q}+{\left({ᶆ}_{k}^{u}\right)}^{q}\right),{\left({ᵰ}_{k}^{l}\right)}^{q}+{\left({ᵰ}_{k}^{u}\right)}^{q}\right]$. Lastly, make the IV-q-RFS decision matrix $Ų={\left({Ŝ}_{k}\right)}_{ɱ\times ȵ}$ is designed by using the q-ROFS concept.

Usually, befit and cost type are two types of attributes:

$$\bar{U_{i}}=\left\{ \begin{array}{c} U_{i}\;\text{margin}\\ U_{i}^{c}\;\text{cost}\; \end{array}\right.$$

Let $U_{i}$ be a margin and $U_{i}^{c}$ be the decision matrix's cost value. There is no need for adjustment when the attributes (margin and cost) are of the exact precise nature. If the margin and cast properties are different, they must be modified.

We suggest a method utilizing the IV-q-ROFAAPWA and IV-q-ROFAAPWG algorithms to elucidate the MAGDM problem, applying the IV-q-ROS concept in this case to select the optimal choice. The following is a list of the steps in this algorithm:

Step 1 Utilize the formula below to calculate the support values:

$$\mathrm{Sup}\left(\beta_{\text{ij}}^{a},\beta_{\text{ij}}^{b}\right)=1-d\left(\beta_{\text{ij}}^{a},\beta_{\text{ij}}^{b}\right)\text{,}$$

In this a,b=1,2,…,p;j=1,2,…,m;i=1,2,…,n which fulfil all environments of support function than can be debated in Eqs. (1), (2), (3), in this $\mathrm{Sup}\left(\beta_{\text{ij}}^{a},\beta_{\text{ij}}^{b}\right)$ displays the distance among dual IV-q-ROFVs $\beta_{\text{ij}}^{a}$ and $\beta_{\text{ij}}^{b}$ and in Definition 3, it is explained.

Step 2 Compute $A\left(\beta_{\text{ij}}^{b}\right)$ value .

$$A\left(\beta_{\text{ij}}^{b}\right)=\sum_{\begin{array}{c} t=1\\ t\neq k \end{array}}^{p}\mathrm{Sup}\left(\beta_{\text{ij}}^{a},\beta_{\text{ij}}^{b}\right)$$

i=1,2,…,n,j=1,2,…,m,b=1,2,…,p;

Step 3 Compute the answer to the WV $\xi_{i}^{k}$ linked through the IV-q-ROFVs $\beta_{\text{ij}}^{b}\text{.}$

$$\xi_{\text{ij}}^{k}=\frac{\gamma_{k}\left(1+A\left(\beta_{\text{ij}}^{k}\right)\right)}{\sum_{i=1}^{p}\gamma_{k}\left(1+A\left(\beta_{\text{ij}}^{k}\right)\right)}\;k=1,2,\ldots ,p$$

Step 4 Using our newly created IV-q-ROFAAPWA and IV-q-ROFAAPWG operators, as shown below, To calculate all the values of ${Ґ}_{i}$.

$$\text{IV}-q-\text{ROFAAPWA}\left({Ґ}_{\text{ij}}^{1},{Ґ}_{\text{ij}}^{2},{Ґ}_{\text{ij}}^{3},\ldots ,{Ґ}_{\text{ij}}^{n}\right)=\left(\left[{\left({ᵯ}_{\text{ij}}^{l}\right)}^{q},{\left({ᵯ}_{\text{ij}}^{u}\right)}^{q}\right],\left[{\left({ᵰ}_{\text{ij}}^{l}\right)}^{q},{\left({ᵰ}_{\text{ij}}^{u}\right)}^{q}\right]\right)$$

$$=\left\langle \left[\begin{array}{c} \sqrt[{q}]{1-e^{-{\left(\sum_{\text{ij}=1}^{p}{\xi_{\text{ij}}\left(-\ln\left(1-{\left({ᵯ}_{\text{ij}}^{l}\right)}^{q}\right)\right)}^{Ή}\right)}^{\frac{1}{Ή}}}},\\ \sqrt[{q}]{1-e^{-{\left(\sum_{\text{ij}=1}^{p}{\xi_{\text{ij}}\left(-\ln\left(1-{\left({ᵯ}_{\text{ij}}^{u}\right)}^{q}\right)\right)}^{Ή}\right)}^{\frac{1}{Ή}}}} \end{array}\right],\left[\begin{array}{c} e^{-{\left(\sum_{\text{ij}=1}^{p}{\xi_{\text{ij}}\left(-\ln\;{ᵰ}_{\text{ij}}^{l}\right)}^{Ή}\right)}^{\frac{1}{Ή}}},\\ e^{-{\left(\sum_{\text{ij}=1}^{p}{\xi_{\text{ij}}\left(-\ln\;{ᵰ}_{\text{ij}}^{u}\right)}^{Ή}\right)}^{\frac{1}{Ή}}} \end{array}\right]\right\rangle$$

and

$$\text{IV}-q-\text{ROFAAPWG}\left({Ґ}_{\text{ij}}^{1},{Ґ}_{\text{ij}}^{2},{Ґ}_{\text{ij}}^{3},\ldots ,{Ґ}_{\text{ij}}^{n}\right)=\left(\left[{\left({ᵯ}_{\text{ij}}^{l}\right)}^{q},{\left({ᵯ}_{\text{ij}}^{u}\right)}^{q}\right],\left[{\left({ᵰ}_{\text{ij}}^{l}\right)}^{q},{\left({ᵰ}_{\text{ij}}^{u}\right)}^{q}\right]\right)$$

$$=\left\langle \left[\begin{array}{c} \sqrt[{q}]{e^{-{\left(\sum_{\text{ij}=1}^{p}{\xi_{\text{ij}}\left(-\ln\;{\left({ᵯ}_{\text{ij}}^{l}\right)}^{q}\right)}^{Ή}\right)}^{\frac{1}{Ή}}}},\\ \sqrt[{q}]{e^{-{\left(\sum_{\text{ij}=1}^{p}{\xi_{\text{ij}}\left(-\ln\;{\left({ᵯ}_{\text{ij}}^{u}\right)}^{q}\right)}^{Ή}\right)}^{\frac{1}{Ή}}}} \end{array}\right],\left[\begin{array}{c} 1-e^{-{\left(\sum_{\text{ij}=1}^{p}{\xi_{\text{ij}}\left(-\ln\left(1-{ᵰ}_{\text{ij}}^{l}\right)\right)}^{Ή}\right)}^{\frac{1}{Ή}}},\\ \;1-e^{-{\left(\sum_{\text{ij}=1}^{p}{\xi_{\text{ij}}\left(-\ln\left(1-{ᵰ}_{\text{ij}}^{u}\right)\right)}^{Ή}\right)}^{\frac{1}{Ή}}} \end{array}\right]\right\rangle$$

Step 5 Utilizing the following formula, one may get the value of $A\left(\beta_{\text{ij}}\right)$.

$$A\left(\beta_{\text{ij}}\right)=\sum_{\begin{array}{c} h=1\\ h\neq j \end{array}}^{n}\mathrm{Sup}\left(\beta_{\text{ij}},\beta_{\text{ih}}\right)i,h,j=1,2,\ldots ,n$$

Step 6 Determining the weight vector's value $\xi_{i}^{k}$ related to the IV-q-ROFVs $\beta_{\text{ij}}^{k}\text{.}$

$$\xi_{\text{ij}}^{k}=\frac{\varpi_{i}\left(1+A\left(\beta_{\text{ij}}^{k}\right)\right)}{\sum_{i=1}^{n}\varpi_{i}\left(1+A\left(\beta_{\text{ij}}^{k}\right)\right)}\;k=1,2,\ldots ,n$$

Step 7 Using the suggested AOs discussed above, completely aggregate the data for each attribute:

$$\text{IV}-q-\text{ROFAAPWA}\left({Ґ}_{i1},{Ґ}_{i2},{Ґ}_{i3},\ldots ,{Ґ}_{\text{in}}\right)=\left(\left[{\left({ᵯ}_{\text{ij}}^{l}\right)}^{q},{\left({ᵯ}_{\text{ij}}^{u}\right)}^{q}\right],\left[{\left({ᵰ}_{\text{ij}}^{l}\right)}^{q},{\left({ᵰ}_{\text{ij}}^{u}\right)}^{q}\right]\right)$$

$$=\left\langle \left[\begin{array}{c} \sqrt[{q}]{1-e^{-{\left(\sum_{\text{ij}=1}^{p}{\xi_{\text{ij}}\left(-\ln\left(1-{\left({ᵯ}_{{Ґ}_{\text{ij}}}^{l}\right)}^{q}\right)\right)}^{Ή}\right)}^{\frac{1}{Ή}}}},\\ \sqrt[{q}]{1-e^{-{\left(\sum_{\text{ij}=1}^{p}{\xi_{\text{ij}}\left(-\ln\left(1-{\left({ᵯ}_{{Ґ}_{\text{ij}}}^{u}\right)}^{q}\right)\right)}^{Ή}\right)}^{\frac{1}{Ή}}}} \end{array}\right],\left[\begin{array}{c} e^{-{\left(\sum_{\text{ij}=1}^{p}{\xi_{\text{ij}}\left(-\ln\;{ᵰ}_{{Ґ}_{\text{ij}}}^{l}\right)}^{Ή}\right)}^{\frac{1}{Ή}}},\\ e^{-{\left(\sum_{\text{ij}=1}^{p}{\xi_{\text{ij}}\left(-\ln\;{ᵰ}_{\text{ij}}^{u}\right)}^{Ή}\right)}^{\frac{1}{Ή}}} \end{array}\right]\right\rangle$$

and

$$\text{IV}-q-\text{ROFAAPWG}\left({Ґ}_{i1},{Ґ}_{i2},{Ґ}_{i3},\ldots ,{Ґ}_{\text{in}}\right)=\left(\left[{\left({ᵯ}_{\text{ij}}^{l}\right)}^{q},{\left({ᵯ}_{\text{ij}}^{u}\right)}^{q}\right],\left[{\left({ᵰ}_{\text{ij}}^{l}\right)}^{q},{\left({ᵰ}_{\text{ij}}^{u}\right)}^{q}\right]\right)$$

$$=\left\langle \left[\begin{array}{c} \sqrt[{q}]{e^{-{\left(\sum_{\text{ij}=1}^{p}{\xi_{\text{ij}}\left(-\ln\;{\left({ᵯ}_{{Ґ}_{\text{ij}}}^{l}\right)}^{q}\right)}^{Ή}\right)}^{\frac{1}{Ή}}}},\\ \sqrt[{q}]{e^{-{\left(\sum_{\text{ij}=1}^{p}{\xi_{\text{ij}}\left(-\ln\;{\left({ᵯ}_{{Ґ}_{\text{ij}}}^{u}\right)}^{q}\right)}^{Ή}\right)}^{\frac{1}{Ή}}}} \end{array}\right],\left[\begin{array}{c} 1-e^{-{\left(\sum_{\text{ij}=1}^{p}{\xi_{\text{ij}}\left(-\ln\left(1-{ᵰ}_{{Ґ}_{\text{ij}}}^{l}\right)\right)}^{Ή}\right)}^{\frac{1}{Ή}}},\\ \;1-e^{-{\left(\sum_{\text{ij}=1}^{p}{\xi_{\text{ij}}\left(-\ln\left(1-{ᵰ}_{{Ґ}_{\text{ij}}}^{u}\right)\right)}^{Ή}\right)}^{\frac{1}{Ή}}} \end{array}\right]\right\rangle$$

Where ij=1,2,…,p.

Step 8 Provide the Ranking of alternatives using the SV formula from Liu et al. [58].

$$S\left(c\right)=\left[{\left({ᵯ}_{i}^{l}\right)}^{q},{\left({ᵯ}_{i}^{u}\right)}^{q}\right]-\left[{\left({ᵰ}_{i}^{l}\right)}^{q},{\left({ᵰ}_{i}^{u}\right)}^{q}\right]$$

Step 9 Finally, organize the different alternatives to illustration first-rate choice.

## 5 A numerical example

The importance of electric cars cannot be overstated where nature's fuel resources will end. One of the essential qualities of an electric vehicle is that its engine has a much better ability to convert the stored electric energy to driving power (kinetic energy) with minimum energy wastage. On the other hand, conventional internal combustion engine vehicles waste more fossil fuel energy in the form of heat energy. The new emerging electric car technology helps eliminate fossil fuel dependency and provides low-cost traveling. Electric cars are friendly to the environment. For example, they cannot produce nitrogen oxide, volatile organic compounds, and noise pollution like conventional vehicles. The rapid growth of electric cars is revolutionizing the automobile industry. Soon, all traditional care will fail to new innovative electric car technology. In this challenging situation, it is a big problem for ordinary humans to select the best electric car. Many well-reputed car production companies are working in the market, such as Tesla in the United States, Nissan in Japan, BMW in Germany, Volkswagen in Germany, Hyundai in South Korea, etc. All these companies claim to provide well-updated technological advancement in their vehicles. So, in this confusing situation, by using our suggested IV-q-ROFAAPWA and IV-q-ROFAAPWG operators, we can solve the MAGDM problem.

Example 3 Now, we discuss a real-life problem of electric car selection using developed IV-q-ROFAAPWA and IV-q-ROFAAPWG AOs. The description of the numerical problem is deliberated certain below:

An example is suggested here for the explanation of this method. Take five cars ${Ų}_{i}=\left({Ų}_{1},{Ų}_{2},{Ų}_{3},{Ų}_{4},{Ų}_{4}\right)$ such as ${Ų}_{1}$ is Tesla ${Ų}_{2}$ is Nissan ${Ų}_{3}$ is BMW ${Ų}_{4}$ is Volkswagen, ${Ų}_{5}$ is Hyundai and choose one from all the companies. We calculate all the providers from four angles $A_{i}=\left(A_{1},A_{2},A_{3},A_{4}\right)$ such as $A_{1}$ is charging speed, $A_{2}$ is quality, $A_{3}$ is production, $A_{4}$ is efficiency and $A_{5}$ is safety features, and ϖ=(0.1,0.4,0.3,0.2) represent the WV of attributes. Take four experts and the WV ${\gamma =\left(0.24,0.35,041\right)}^{T}$. ${\left[R_{ij}^{k}\right]}_{5\times 4}={\left(b_{ij}^{k}\right)}_{5\times 4}$ show the DM given in Table 1, Table 2, Table 3. In the form of IV-q-ROF information. Our ambition is to choose the best car from all the alternative companies. All procedures performed in this study were in accordance with the ethical standards of the university. Ethical clearance and approval were granted by Riphah International University Lahore. Informed consent: Informed consent was obtained from all individual participants included in the study

**Table 1:** (IV-q-ROFS decision matrix $A^{1}$).

	${Ų}_{1}$	${Ų}_{2}$	${Ų}_{3}$	${Ų}_{4}$
$A_{1}$	$\left(\begin{array}{c} \left[0.20,0.40\right]\text{,}\\ \left[0.40,0.50\right] \end{array}\right)$	$\left(\begin{array}{c} \left[0.30,0.50\right]\text{,}\\ \left[0.31,0.45\right] \end{array}\right)$	$\left(\begin{array}{c} \left[0.25,0.46\right]\text{,}\\ \left[0.32,0.56\right] \end{array}\right)$	$\left(\begin{array}{c} \left[0.36,0.56\right]\text{,}\\ \left[0.49,0.53\right] \end{array}\right)$
$A_{2}$	$\left(\begin{array}{c} \left[0.23,0.42\right]\text{,}\\ \left[0.38,0.60\right] \end{array}\right)$	$\left(\begin{array}{c} \left[0.29,0.32\right]\text{,}\\ \left[0.39,0.76\right] \end{array}\right)$	$\left(\begin{array}{c} \left[0.28,0.53\right]\text{,}\\ \left[0.36,0.73\right] \end{array}\right)$	$\left(\begin{array}{c} \left[0.12,0.61\right]\text{,}\\ \left[0.40,0.77\right] \end{array}\right)$
$A_{2}$	$\left(\begin{array}{c} \left[0.22,0.45\right]\text{,}\\ \left[0.39,0.63\right] \end{array}\right)$	$\left(\begin{array}{c} \left[0.41,0.42\right]\text{,}\\ \left[0.30,0.62\right] \end{array}\right)$	$\left(\begin{array}{c} \left[0.34,0.53\right]\text{,}\\ \left[0.36,0.66\right] \end{array}\right)$	$\left(\begin{array}{c} \left[0.32,0.53\right]\text{,}\\ \left[0.48,0.54\right] \end{array}\right)$
$A_{4}$	$\left(\begin{array}{c} \left[0.23,0.48\right]\text{,}\\ \left[0.36,0.52\right] \end{array}\right)$	$\left(\begin{array}{c} \left[0.42,0.52\right]\text{,}\\ \left[0.32,0.62\right] \end{array}\right)$	$\left(\begin{array}{c} \left[0.36,0.61\right]\text{,}\\ \left[0.35,0.64\right] \end{array}\right)$	$\left(\begin{array}{c} \left[0.49,0.52\right]\text{,}\\ \left[0.57,0.59\right] \end{array}\right)$
$A_{5}$	$\left(\begin{array}{c} \left[0.32,0.42\right]\text{,}\\ \left[0.43,0.56\right] \end{array}\right)$	$\left(\begin{array}{c} \left[0.43,0.46\right]\text{,}\\ \left[0.31,0.56\right] \end{array}\right)$	$\left(\begin{array}{c} \left[0.50,0.43\right]\text{,}\\ \left[0.41,0.54\right] \end{array}\right)$	$\left(\begin{array}{c} \left[0.51,0.65\right]\text{,}\\ \left[0.31,0.32\right] \end{array}\right)$

**Table 2:** (IV-q-ROFS decision matrix $A^{2}$).

	${Ų}_{1}$	${Ų}_{2}$	${Ų}_{3}$	${Ų}_{4}$
$A_{1}$	$\left(\begin{array}{c} \left[0.34,0.54\right]\text{,}\\ \left[0.26,0.71\right] \end{array}\right)$	$\left(\begin{array}{c} \left[0.45,0.65\right]\text{,}\\ \left[0.26,0.56\right] \end{array}\right)$	$\left(\begin{array}{c} \left[0.42,0.62\right]\text{,}\\ \left[0.32,0.72\right] \end{array}\right)$	$\left(\begin{array}{c} \left[0.43,0.56\right]\text{,}\\ \left[0.45,0.56\right] \end{array}\right)$
$A_{2}$	$\left(\begin{array}{c} \left[0.24,0.44\right]\text{,}\\ \left[0.38,0.68\right] \end{array}\right)$	$\left(\begin{array}{c} \left[0.29,0.39\right]\text{,}\\ \left[0.45,0.71\right] \end{array}\right)$	$\left(\begin{array}{c} \left[0.32,0.42\right]\text{,}\\ \left[0.41,0.62\right] \end{array}\right)$	$\left(\begin{array}{c} \left[0.13,0.32\right]\text{,}\\ \left[0.43,0.53\right] \end{array}\right)$
$A_{2}$	$\left(\begin{array}{c} \left[0.21,0.53\right]\text{,}\\ \left[0.45,0.71\right] \end{array}\right)$	$\left(\begin{array}{c} \left[0.33,0.54\right]\text{,}\\ \left[0.31,0.61\right] \end{array}\right)$	$\left(\begin{array}{c} \left[0.50,0.65\right]\text{,}\\ \left[0.37,0.72\right] \end{array}\right)$	$\left(\begin{array}{c} \left[0.45,0.52\right]\text{,}\\ \left[0.42,0.46\right] \end{array}\right)$
$A_{4}$	$\left(\begin{array}{c} \left[0.22,0.33\right]\text{,}\\ \left[0.36,0.78\right] \end{array}\right)$	$\left(\begin{array}{c} \left[0.31,0.41\right]\text{,}\\ \left[0.32,0.42\right] \end{array}\right)$	$\left(\begin{array}{c} \left[0.16,0.72\right]\text{,}\\ \left[0.45,0.4s6\right] \end{array}\right)$	$\left(\begin{array}{c} \left[0.46,0.77\right]\text{,}\\ \left[0.34,0.61\right] \end{array}\right)$
$A_{5}$	$\left(\begin{array}{c} \left[0.35,0.45\right]\text{,}\\ \left[0.22,0.64\right] \end{array}\right)$	$\left(\begin{array}{c} \left[0.43,0.53\right]\text{,}\\ \left[0.31,0.48\right] \end{array}\right)$	$\left(\begin{array}{c} \left[0.45,0.61\right]\text{,}\\ \left[0.44,0.68\right] \end{array}\right)$	$\left(\begin{array}{c} \left[0.47,0.67\right]\text{,}\\ \left[0.12,0.39\right] \end{array}\right)$

**Table 3:** (IV-q-ROFS decision matrix $A^{3}$).

	${Ų}_{1}$	${Ų}_{2}$	${Ų}_{3}$	${Ų}_{4}$
$A_{1}$	$\left(\begin{array}{c} \left[0.36,0.48\right]\text{,}\\ \left[0.38,0.71\right] \end{array}\right)$	$\left(\begin{array}{c} \left[0.57,0.61\right]\text{,}\\ \left[0.39,0.45\right] \end{array}\right)$	$\left(\begin{array}{c} \left[0.56,0.62\right]\text{,}\\ \left[0.32,0.45\right] \end{array}\right)$	$\left(\begin{array}{c} \left[0.27,0.74\right]\text{,}\\ \left[0.63,0.67\right] \end{array}\right)$
$A_{2}$	$\left(\begin{array}{c} \left[0.25,0.72\right]\text{,}\\ \left[0.45,0.72\right] \end{array}\right)$	$\left(\begin{array}{c} \left[0.34,0.48\right]\text{,}\\ \left[0.61,0.65\right] \end{array}\right)$	$\left(\begin{array}{c} \left[0.52,0.57\right]\text{,}\\ \left[0.36,0.61\right] \end{array}\right)$	$\left(\begin{array}{c} \left[0.12,0.18\right]\text{,}\\ \left[0.45,0.49\right] \end{array}\right)$
$A_{2}$	$\left(\begin{array}{c} \left[0.24,0.62\right]\text{,}\\ \left[0.43,0.68\right] \end{array}\right)$	$\left(\begin{array}{c} \left[0.44,0.58\right]\text{,}\\ \left[0.56,0.33\right] \end{array}\right)$	$\left(\begin{array}{c} \left[0.50,0.51\right]\text{,}\\ \left[0.36,0.53\right] \end{array}\right)$	$\left(\begin{array}{c} \left[0.34,0.45\right]\text{,}\\ \left[0.56,0.69\right] \end{array}\right)$
$A_{4}$	$\left(\begin{array}{c} \left[0.25,0.45\right]\text{,}\\ \left[0.31,0.72\right] \end{array}\right)$	$\left(\begin{array}{c} \left[0.45,0.51\right]\text{,}\\ \left[0.21,0.25\right] \end{array}\right)$	$\left(\begin{array}{c} \left[0.16,0.31\right]\text{,}\\ \left[0.35,0.46\right] \end{array}\right)$	$\left(\begin{array}{c} \left[0.38,0.59\right]\text{,}\\ \left[0.56,0.69\right] \end{array}\right)$
$A_{5}$	$\left(\begin{array}{c} \left[0.34,0.46\right]\text{,}\\ \left[0.25,0.61\right] \end{array}\right)$	$\left(\begin{array}{c} \left[0.29,0.43\right]\text{,}\\ \left[0.35,0.39\right] \end{array}\right)$	$\left(\begin{array}{c} \left[0.55,0.71\right]\text{,}\\ \left[0.41,0.48\right] \end{array}\right)$	$\left(\begin{array}{c} \left[0.46,0.55\right]\text{,}\\ \left[0.61,0.71\right] \end{array}\right)$

## 6 Evaluation steps

Keynote assessment stepladders are shown as particular following:

Step 1 Initially, Collect information from anonymous decision-makers using FVs and allow the weights to decision-makers such as $\left(\;D_{1},D_{2},D_{3}\right)$ is (0.24,0.35,0.41) respectively. We also assign the weights to our considered attributes, such as 0.30 for the risk analysis $\left(A_{1}\right)$; 0.20 for historical return analysis $\left(A_{2}\right)$; 0.24 for the interest of people $\left(A_{3}\right)$; 0.26 for competition in the marketplace $\left(A_{4}\right)$. Table 1, Table 2, Table 3 contain a collection of FVs information.

Step 2 Utilizing the proposed IV-q-ROFAAPWA and IV-q-ROFAAPWG, aggregate the fuzzy information by taking the parameters q=3 and Ή=1. Table 4 displays the AOs and compiled data.

**Table 4:** Aggregation outcomes.

	IV-q-ROFAAPWA	IV-q-ROFAAPWG
${Ų}_{1}$	$\left(\begin{array}{c} \left[0.1748,0.2615\right]\text{,}\\ \left[0.0128,0.0117\right] \end{array}\right)$	$\left(\begin{array}{c} \left[0.6083,0.6034\right]\text{,}\\ \left[0.2287,0.3949\right] \end{array}\right)$
${Ų}_{2}$	$\left(\begin{array}{c} \left[0.1385,0.1972\right]\text{,}\\ \left[0.0134,0.0277\right] \end{array}\right)$	$\left(\begin{array}{c} \left[0.5866,0.6276\right]\text{,}\\ \left[0.2545,0.3978\right] \end{array}\right)$
${Ų}_{3}$	$\left(\begin{array}{c} \left[0.1701,0.2186\right]\text{,}\\ \left[0.0139,0.0239\right] \end{array}\right)$	$\left(\begin{array}{c} \left[0.6068,0.6429\right]\text{,}\\ \left[0.2513,0.3636\right] \end{array}\right)$
${Ų}_{4}$	$\left(\begin{array}{c} \left[0.1602,0.3244\right]\text{,}\\ \left[0.0122,0.0022\right] \end{array}\right)$	$\left(\begin{array}{c} \left[0.5958,0.4873\right]\text{,}\\ \left[0.2278,0.4964\right] \end{array}\right)$
${Ų}_{5}$	$\left(\begin{array}{c} \left[0.1902,0.2205\right]\text{,}\\ \left[0.0115,0.0195\right] \end{array}\right)$	$\left(\begin{array}{c} \left[0.6202,0.6416\right]\text{,}\\ \left[0.2019,0.3234\right] \end{array}\right)$

**Fig. 1:**
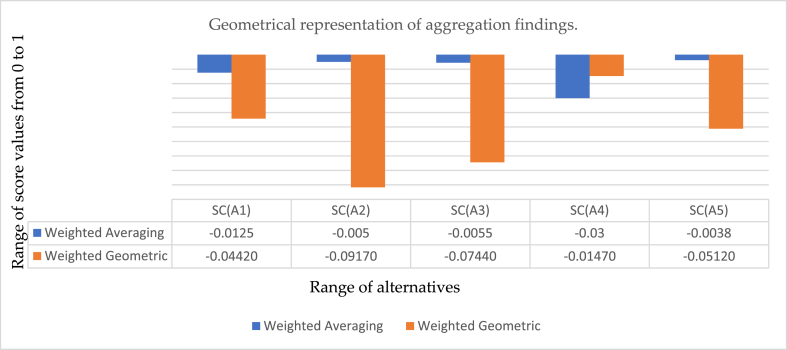
Provides a precise geometrical representation of Table 5.

Step 3 In arrange to find which choice is better, use the SV formula from Definition 2. Table 5 displays the SV results. In Fig. 1, our argument is also expressed geometrically.

**Table 5:** SV of aggregated outcomes.

	IV-q-ROFAAPWA	IV-q-ROFAAPWG
${Ų}_{1}$	−0.0125	−0.0442
${Ų}_{2}$	−0.0050	−0.0917
${Ų}_{3}$	−0.0055	−0.0744
${Ų}_{4}$	−0.0300	−0.0147
${Ų}_{5}$	−0.0038	−0.0512

For better understanding, we presented our aggregation findings in the geometrical format, as shown in Fig. 1. The blue line represents the results obtained by WA operators. In contrast, orange lines denote aggregated outcomes obtained through WG operators. It is also clearly observed that when we use IV-q-ROFAAPWA operators ${Ų}_{5}$ is the best alternative. When we apply IV-q-ROFAAPWG operators to the given information, then ${Ų}_{4}$ is the best option.

The horizontal line shows the range of score values, and the vertical line shows the range of alternatives. Also, we noticed that the blue lines represent the IV-q-ROFAAPWA operators' ranking order, while the orange lines define the IV-q-ROFAAPWG operators' ranking order.

Step 4 Putting the calculating' all values in order of their rightness for their SV. When applying the IV-q-ROFAAPWA operator, it is found that ${Ų}_{5}$ is the better selection among all the companies, but ${Ų}_{4}$ is the better decision between all the companies when using the IV-qROFAAPWG operator. In Table 6, the ranking arrangement is displayed.

**Table 6:** Ranking of SVs.

	Ordering
IV-q-ROFAAPWA	${Ų}_{5}>{Ų}_{2}>{Ų}_{3}>{Ų}_{1}>{Ų}_{4}$
IV-q-ROFAAPWG	${Ų}_{4}>{Ų}_{1}>{Ų}_{5}>{Ų}_{3}>{Ų}_{2}$

## 7 Sensitive analysis of parameters

The sensitivity analysis of the parameters utilized in the suggested AOs is covered in this section. This analysis demonstrates how the two parameters q and Ή affect the produced AOs. We use graphical representation to convey our astute observations and the variable effects of the parameters on ranking order.

### 7.1 Effect of Ή

From the suggested real-life example, it is seen that change in Ή by the decision makers causes the difference in the sequence of ranking order. For this, when we take Ή=1,3,5,7,11,…n and in the whole procedure, we take q=3. Then, Table 7 shows changes to the IV-q-ROFAAPWA operator's ranking order. Table 7 shows what happens when substituting Ή=1,3,5,7,11,…n for the IV-q-ROFAAPWG operator.

**Table 7:** Ranking of the sequence of IV-q-ROFAAPWA by changing in parameter Ή.

Ordering the SV using IV-q-ROFAAPWA			
Ή	Ordering	Ή	Ordering
1	${Ų}_{5}>{Ų}_{3}>{Ų}_{2}>{Ų}_{1}>{Ų}_{4}$	2	Result not found
3	${Ų}_{1}>{Ų}_{5}>{Ų}_{2}>{Ų}_{3}>{Ų}_{4}$	4	Result not found
5	${Ų}_{1}>{Ų}_{5}>{Ų}_{3}>{Ų}_{2}>{Ų}_{4}$	6	Result not found
7	${Ų}_{5}>{Ų}_{1}>{Ų}_{3}>{Ų}_{2}>{Ų}_{4}$	8	Result not found
9	${Ų}_{5}>{Ų}_{1}>{Ų}_{3}>{Ų}_{2}>{Ų}_{4}$	10	Result not found
11	${Ų}_{5}>{Ų}_{3}>{Ų}_{2}>{Ų}_{1}>{Ų}_{4}$	12	Result not found

**Fig. 2:**
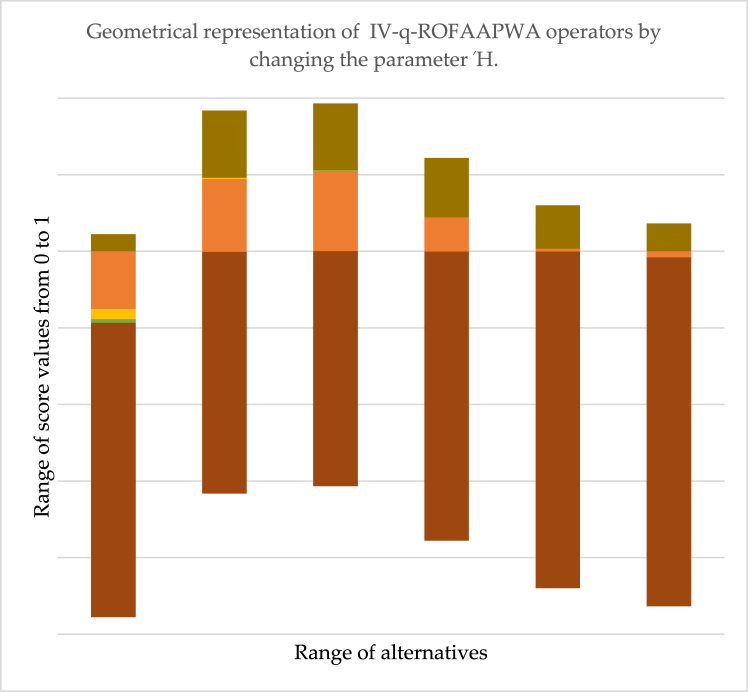
The geometrical representation of the IV-q-ROFAAPWA operator's score value with variation in Ή.

Table 7 presents the change in aggregation findings by changing the parametric value of Ή in proposed IV-q-ROFAAPWA operators. It is noticed that when we take the value of Ή is 1,3,5,7,9, then significant changes occur in results; on the other hand, we found that when we place Ή=11 and all upcoming value odd values, then there will be no changes appear in aggregated results. We also noticed that no answer is obtained if Ή is assumed to be an even number. Graphical representation of Table 7 can be seen in Fig. 2.

**Fig. 3:**
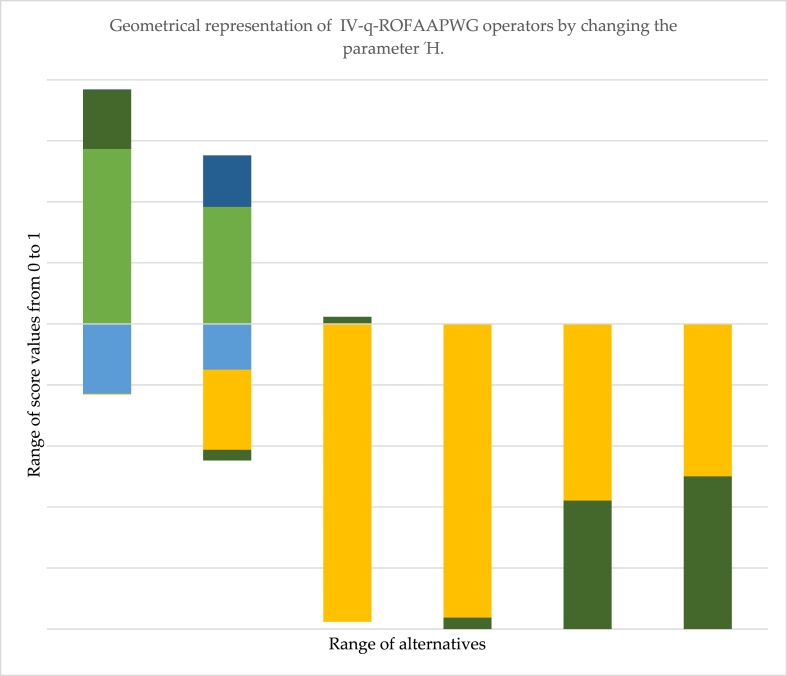
The geometrical representation of the IV-q-ROFAAPWG operator's score value with variation in Ή.

Table 8 shows the possible changes that occurred by changing the parametric value during data aggregation by applying IV-q-ROFAAPWG operators. It is significant that when we take Ή=1,3,5,7,9, then the ranking sequence of aggregated information varies by the variation of the parametric value of Ή. On the other side, we observed that when we tale Ή=11 and for further all odd deals, the ranking order will remain the same, which means that when Ή=11 is considered a stability point for IV-q-ROFAAPWG operators. Also, we found that no answer is obtained when we assume Ή is an even number. A graphical representation of Table 8 is given in Fig. 3.

**Table 8:** Ranking of the sequence of IV-q-ROFAAPWG by changing in parameter Ή.

Ordering the SV using IV-q-ROFAAPWG			
Ή	Ordering	Ή	Ordering
1	${Ų}_{1}>{Ų}_{4}>{Ų}_{5}>{Ų}_{3}>{Ų}_{2}$	2	Result not found
3	${Ų}_{1}>{Ų}_{5}>{Ų}_{4}>{Ų}_{2}>{Ų}_{3}$	4	Result not found
5	${Ų}_{4}>{Ų}_{5}>{Ų}_{2}>{Ų}_{1}>{Ų}_{3}$	6	Result not found
7	${Ų}_{5}>{Ų}_{1}>{Ų}_{2}>{Ų}_{4}>{Ų}_{3}$	8	Result not found
9	${Ų}_{5}>{Ų}_{1}>{Ų}_{2}>{Ų}_{4}>{Ų}_{3}$	10	Result not found
11	${Ų}_{5}>{Ų}_{1}>{Ų}_{2}>{Ų}_{3}>{Ų}_{4}$	12	Result not found

The horizontal line shows the range of score values, and the vertical line shows the range of alternatives. The above figure shows the graphical interpretation of information discussed in Table 7 from the diagrams, we quickly observed that when we take Ή=1, then ${Ų}_{5}$ be the best alternative, while when Ή=3,5 ${Ų}_{1}$ be the best option. However, when we take Ή=7,9,11 then, the finest result is ${Ų}_{5}$.

The Fig. 3. Shows the variational effects of parameter Ή on the proposed IV-q-ROFAAPWG operator. The horizontal line shows the range of score values, and the vertical line shows the range of alternatives. The information from the above diagram is as follows: when we take the value of parameter Ή=1,3, then the best raking alternative is ${Ų}_{1}$ while when we take Ή=5, the finest optimum result is ${Ų}_{5}$. However, when we place the value of parameter Ή=7,9,11, then the best resultant is ${Ų}_{5}$ by utilizing the developed IV-q-ROFAAPWG operator.

### 7.2 Effect of q

The operators, IV-q-RPFAAPWA and IV-q-ROFAAPWG, are observed for q=3 and the IV-q-ROF information. It can be shown that changing the value of q has no impact on the arrangement of the same options. Therefore, for the IV-q-RPFAAPWA and IV-q-ROFAAPWG operators, we can modify the value of q. Table 9, Table 10 show the varying properties of q for the IV-q-RPFAAPWA and IV-q-ROFAAPWG operators.

**Table 9:** The ranking sequence of SVs by varying q in IV-q-ROFAAPWA.

q	Ordering
3	${Ų}_{5}>{Ų}_{3}>{Ų}_{2}>{Ų}_{1}>{Ų}_{4}$
4	${Ų}_{5}>{Ų}_{3}>{Ų}_{2}>{Ų}_{1}>{Ų}_{4}$
5	${Ų}_{5}>{Ų}_{3}>{Ų}_{2}>{Ų}_{1}>{Ų}_{4}$
6	${Ų}_{5}>{Ų}_{3}>{Ų}_{2}>{Ų}_{1}>{Ų}_{4}$
7	${Ų}_{5}>{Ų}_{3}>{Ų}_{2}>{Ų}_{1}>{Ų}_{4}$
8	${Ų}_{5}>{Ų}_{3}>{Ų}_{2}>{Ų}_{1}>{Ų}_{4}$
**9**	${Ų}_{5}>{Ų}_{3}>{Ų}_{2}>{Ų}_{1}>{Ų}_{4}$

**Table 10:** The ranking sequence of SVs by varying q in IV-q-ROFAAPWG.

q	Ordering
3	${Ų}_{1}>{Ų}_{4}>{Ų}_{5}>{Ų}_{3}>{Ų}_{2}$
4	${Ų}_{4}>{Ų}_{1}>{Ų}_{5}>{Ų}_{3}>{Ų}_{2}$
5	${Ų}_{4}>{Ų}_{1}>{Ų}_{5}>{Ų}_{3}>{Ų}_{2}$
6	${Ų}_{4}>{Ų}_{1}>{Ų}_{5}>{Ų}_{3}>{Ų}_{2}$
7	${Ų}_{4}>{Ų}_{1}>{Ų}_{5}>{Ų}_{3}>{Ų}_{2}$
8	${Ų}_{4}>{Ų}_{1}>{Ų}_{5}>{Ų}_{3}>{Ų}_{2}$
**9**	${Ų}_{1}>{Ų}_{4}>{Ų}_{5}>{Ų}_{3}>{Ų}_{2}$

**Fig. 4:**
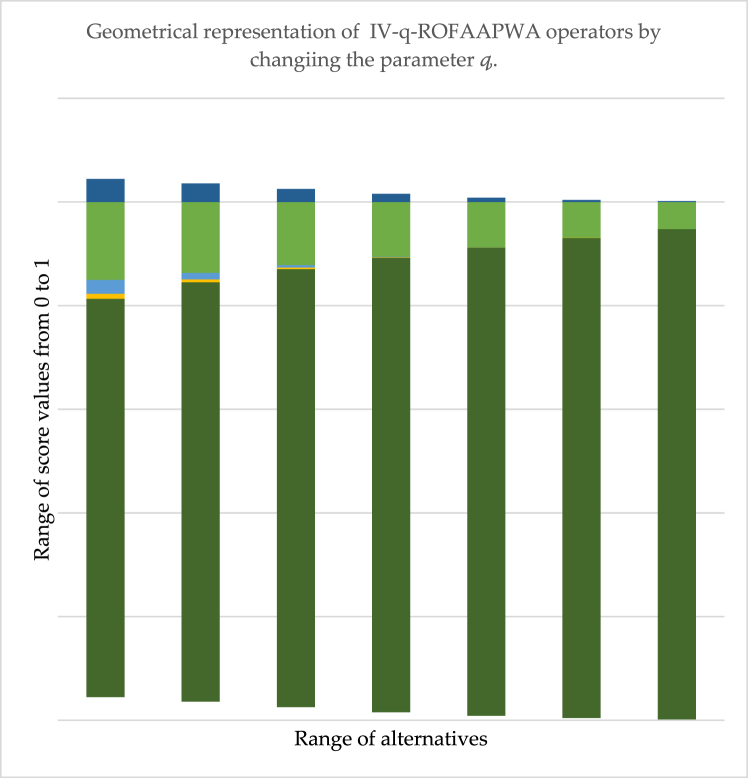
The geometrical representation of the IV-q-ROFAAPWA operator's score value with change by q.

Table 9 shows the effect of parameter q on our suggested method. It is noticed that when we vary the value of q in the IV-q-ROFAAPWA operator, then there is no effect on ranking results. The ranking sequence remains the same for q-ROFS information. Also, we found that there will be no effect of even or odd values on our developed operator. The geometrical depreciation of Table 9 it is provided in Fig. 4.

Fig. 4 represents the information presented in Table 9. The horizontal line shows the range of score values, and the vertical line shows the range of alternatives. It is noted from the above diagram there is no effect on ranking alternatives by taking the variation of parameter q in the IV-q-ROFAAPWA operator. For all value of q=3,4,5,…,n the ranking sequence will remain same such as ${Ų}_{5}>{Ų}_{3}>{Ų}_{2}>{Ų}_{1}>{Ų}_{4}$ and ${Ų}_{5}$ is the best option among all the possibilities.

By adjusting the value of q in the developed IV-q-ROFAAPWG operator, we can observe from the information the ranking sequence remains unchanged. However, as the parameter q is increased, the score values rapidly decline, but the ranking order will remain the same.

**Fig. 5:**
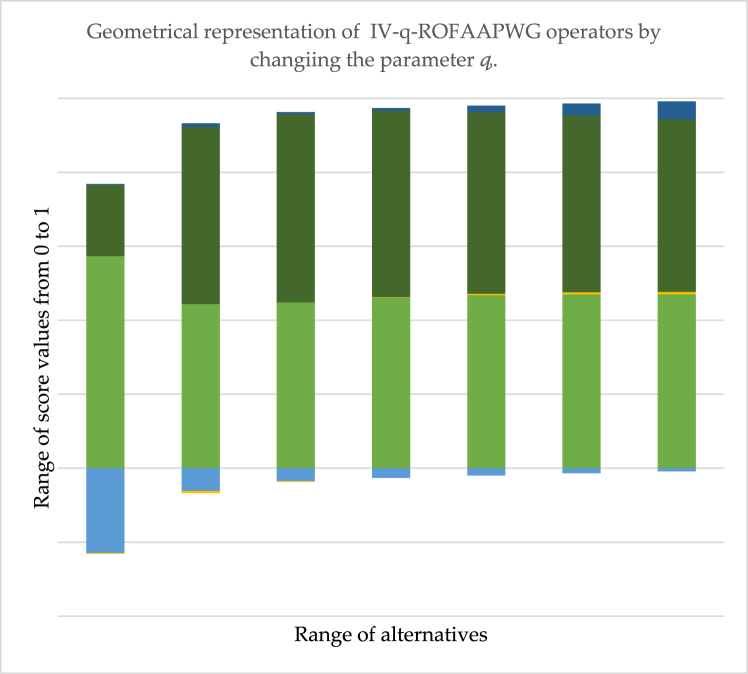
The geometrical representation of the IV-q-ROFAAPWG operator's score value with change by q.

Fig. 5 illustrate the information provided in Table 10. The horizontal line shows the range of score values, and the vertical line shows the range of alternatives. It is noted from the above figure there is no effect on the ranking sequence by taking the variation of parameter q in the proposed IV-q-ROFAAPWG operator. For all value of q=3,4,5,…,n the ranking order will remain same such as ${Ų}_{5}>{Ų}_{3}>{Ų}_{2}>{Ų}_{1}>{Ų}_{4}$ and ${Ų}_{5}$ is the finest alternative of all the options.

## 8 Comparative study

In this part, we can compare our suggested IV-q-ROFAAPWA and IV-q-ROFAAPWG operators by other present AOs, for example, interval-valued q-ROF arithmetic weighted averaging (WA) (IV-q-ROFAWA) and interval-valued q-ROF arithmetic weighted geometric (WG) (IV-q-ROFAWG) operators discussed on [59] and interval-valued q-ROF Hamacher WA (IV-q-ROFHWA) and interval-valued q-ROF Hamacher WG (IV- q-ROF HWG) operators presented in Ref. [60]. The concept of interval-valued q-ROF Dombi WA (IV-q-ROFDWA) and interval-valued q-ROF Dombi WG (IV-q-ROFDWG) operators was proposed in Ref. [61]. Many other AOs can deal with interval-valued q-ROF information, for example, interval-valued IF WA (IV-IFWA) and interval-valued IF WG (IV-IFWG) operators developed in Ref. [62] and the theory of interval-valued IF Hamacher WA (IV-IFHWA) and interval-valued IF Hamacher WG (IV-IFHWG) operators diagnosed in Ref. [38]. The idea of interval-valued PyF Hamacher WA (IV-PyFHWA) and interval-valued PyF Hamacher WG (IV-PyFHWG) operators given in Ref. [63] and the concept of interval-valued PyF Dombi WA (IV-PyFDWA) and interval-valued PyF Domi WG (IV-PyFDWG) operators provided in Ref. [64]. Due to structural shortcomings, these stated AOs cannot handle q-ROFS data. The comparasion of above discussed AOs is provided in Table 11.

**Table 11:** Comparative study.

Methods	Operators	Ranking order
Proposed operators	IV-q-ROFAAPWA (q=3,Ή=1)	${Ų}_{5}>{Ų}_{2}>{Ų}_{3}>{Ų}_{1}>{Ų}_{4}$
	IV-q-ROFAAPWG (q=3,Ή=1)	${Ų}_{4}>{Ų}_{1}>{Ų}_{5}>{Ų}_{3}>{Ų}_{2}$
[59]	IV-q-ROFAWA	${Ų}_{5}>{Ų}_{1}>{Ų}_{3}>{Ų}_{4}>{Ų}_{2}$
	IV-q-ROFAWG	${Ų}_{5}>{Ų}_{3}>{Ų}_{4}>{Ų}_{2}>{Ų}_{1}$
[60]	IV-q-ROFHWA	${Ų}_{1}>{Ų}_{5}>{Ų}_{4}>{Ų}_{3}>{Ų}_{2}$
	IV-q-ROFHWG	${Ų}_{5}>{Ų}_{1}>{Ų}_{3}>{Ų}_{2}>{Ų}_{4}$
[61]	IV-q-ROFDWA	${Ų}_{1}>{Ų}_{5}>{Ų}_{3}>{Ų}_{4}>{Ų}_{2}$
	IV-q-ROFDWG	${Ų}_{1}>{Ų}_{5}>{Ų}_{3}>{Ų}_{4}>{Ų}_{2}$
[62]	IV-IFWA IV-IFWG	✗
[38]	IV-IFHWA IV-IFHWG	✗
[63]	IV-PyFHWA IV-PyFHWG	✗
[64]	IV-PyFDWA IV-PyFDWG	✗

**Fig. 6:**
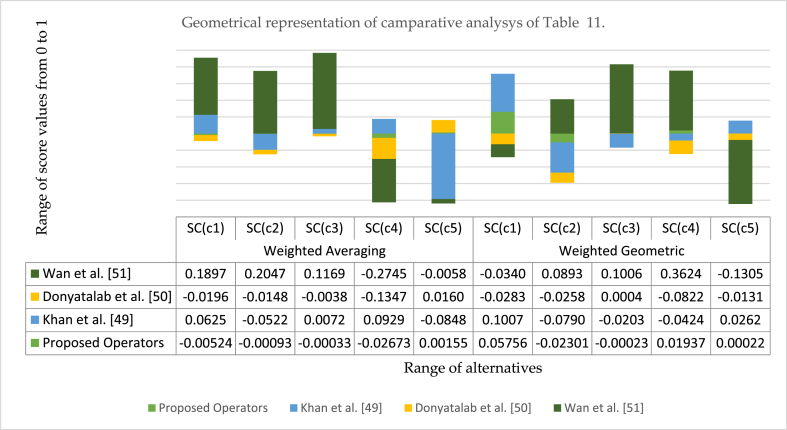
The geometrical representation of comparison with existing AOs.

For convenience, the pictorial representation of Table 11 is presented in Fig. 6. A comparison of our suggested approach with existing AOs was observed, presented in Refs. [[59], [60], [61]]. And some AOs are not able to deal with interval-valued q-ROF data like frameworks are discussed in Refs. [38,[62], [63], [64]].

The diagram represents the graphical view of Table 11. The horizontal line shows the range of score values, and the vertical line shows the range of alternatives. We compare our diagnosed IV-q-ROFAAPWA and IV-q-ROFAAPWG operators with other existing AOs. For example, AOs presented in Refs. [[59], [60], [61], [62]]. It is noticed from the graphical view. ${Ų}_{1}$ be the finest option by using AOs developed in Ref. [62], while ${Ų}_{1}$ be the best option by applying AOs proposed in Ref. [60] and ${Ų}_{5}$ be the best alternative using the AO operator submitted in Ref. [59].

## 9 Results and discussions

This section discusses the novelty of IV-q-ROFS, AATN, AATCN, and PAO concepts. Also shows how proposed operators give more freedom to decision-makers for data aggregation in a precise way, where many fuzzy environments fail to deal with vague information.

The topic of decision-making sciences is always trending and interesting for mathematicians. In this regard, many mathematicians and researchers proposed fuzzy frameworks for data aggregation with preciseness. The thought of interval-valued FS gives a more generalized form of many simple FS because it can discuss MD's lower and upper terms; instead, FS only discusses the thought of MD. By following the same scenario, the idea of IV-q-ROFS can deal with information given in the form of IV-FS, IV-IFS, and IV-PyFS, and it is considered the superior shape of existing structures. Using the idea of IV-q-ROFS, AA operational rules, and a combination of PAOs, the novel approach of IV-q-ROFAAPWA and IV-q-ROFAAPWG operators is proposed. Also, investigate some necessary axioms of AOs such as idempotency, monotonicity, and boundedness. To show the worth of the developed technique provided the algorithm based on the MAGDM methodology and solve the real-life car selection problem.

The car selection problem is one of the trending issues nowadays. A good car is considered a reflection of your personality and represents your life status. So, in the age of technology, it is challenging to select the most appropriate car within a given budget. In this regard, many mathematicians present different thoughts and techniques for selection procedures. However, our diagnosed theory applies to previously existing structures such as IV-FS, IV-IFS, and IV-PyFS. In the developed approach, the power qth gives a large amount of freedom to decision-makers for data aggregation. The consequences of the proposed AOs are discussed as follows:

### 9.1 Consequences of proposed methodology

Some interesting facts about diagnosis theory are given as follows:

$$\text{IV}-q-\text{ROFAAPWA}\left({Ґ}_{i1},{Ґ}_{i2},{Ґ}_{i3},\ldots ,{Ґ}_{\text{in}}\right)=\left(\left[{\left({ᵯ}_{\text{ij}}^{l}\right)}^{q},{\left({ᵯ}_{\text{ij}}^{u}\right)}^{q}\right],\left[{\left({ᵰ}_{\text{ij}}^{l}\right)}^{q},{\left({ᵰ}_{\text{ij}}^{u}\right)}^{q}\right]\right)$$

$$=\left\langle \left[\begin{array}{c} \sqrt[{q}]{1-e^{-{\left(\sum_{\text{ij}=1}^{p}{\xi_{\text{ij}}\left(-\ln\left(1-{\left({ᵯ}_{{Ґ}_{\text{ij}}}^{l}\right)}^{q}\right)\right)}^{Ή}\right)}^{\frac{1}{Ή}}}},\\ \sqrt[{q}]{1-e^{-{\left(\sum_{\text{ij}=1}^{p}{\xi_{\text{ij}}\left(-\ln\left(1-{\left({ᵯ}_{{Ґ}_{\text{ij}}}^{u}\right)}^{q}\right)\right)}^{Ή}\right)}^{\frac{1}{Ή}}}} \end{array}\right],\left[\begin{array}{c} e^{-{\left(\sum_{\text{ij}=1}^{p}{\xi_{\text{ij}}\left(-\ln\;{ᵰ}_{{Ґ}_{\text{ij}}}^{l}\right)}^{Ή}\right)}^{\frac{1}{Ή}}},\\ e^{-{\left(\sum_{\text{ij}=1}^{p}{\xi_{\text{ij}}\left(-\ln\;{ᵰ}_{\text{ij}}^{u}\right)}^{Ή}\right)}^{\frac{1}{Ή}}} \end{array}\right]\right\rangle$$

and

$$\text{IV}-q-\text{ROFAAPWG}\left({Ґ}_{i1},{Ґ}_{i2},{Ґ}_{i3},\ldots ,{Ґ}_{\text{in}}\right)=\left(\left[{\left({ᵯ}_{\text{ij}}^{l}\right)}^{q},{\left({ᵯ}_{\text{ij}}^{u}\right)}^{q}\right],\left[{\left({ᵰ}_{\text{ij}}^{l}\right)}^{q},{\left({ᵰ}_{\text{ij}}^{u}\right)}^{q}\right]\right)$$

$$=\left\langle \left[\begin{array}{c} \sqrt[{q}]{e^{-{\left(\sum_{\text{ij}=1}^{p}{\xi_{\text{ij}}\left(-\ln\;{\left({ᵯ}_{{Ґ}_{\text{ij}}}^{l}\right)}^{q}\right)}^{Ή}\right)}^{\frac{1}{Ή}}}},\\ \sqrt[{q}]{e^{-{\left(\sum_{\text{ij}=1}^{p}{\xi_{\text{ij}}\left(-\ln\;{\left({ᵯ}_{{Ґ}_{\text{ij}}}^{u}\right)}^{q}\right)}^{Ή}\right)}^{\frac{1}{Ή}}}} \end{array}\right],\left[\begin{array}{c} 1-e^{-{\left(\sum_{\text{ij}=1}^{p}{\xi_{\text{ij}}\left(-\ln\left(1-{ᵰ}_{{Ґ}_{\text{ij}}}^{l}\right)\right)}^{Ή}\right)}^{\frac{1}{Ή}}},\\ \;1-e^{-{\left(\sum_{\text{ij}=1}^{p}{\xi_{\text{ij}}\left(-\ln\left(1-{ᵰ}_{{Ґ}_{\text{ij}}}^{u}\right)\right)}^{Ή}\right)}^{\frac{1}{Ή}}} \end{array}\right]\right\rangle$$

It is noticed that when we place the value of NMD at zero and q=1, the proposed AOs are reduced into the interval-valued FS (IVFS) structure. Also, when we take q=1, the defined system is turned into the interval valued-IFS (IVIFS) environment. However, by taking the value of q=2, the diagnosed theory is converted into the shape of interval-valued PyFS (IVPyFS). Hence, it is concluded that our suggested work is suitable for already existing fuzzy frameworks such as IVFS, IVIFS, and IVPyFS. Also, our proposed work is more generalized.

## 10 Conclusion

The arrangement of this article is based on the IV-q-ROFS information. The model of IV-q-ROFS provides more flexibility to decision-makers by enlarging the space of MD and NMD in terms of intervals. The IV-q-ROFS is the generalized form of IVFS, IVIFS and IVPyFSs. Hence, it is concluded that our proposed results also can aggregate data, which is given in the form of IFVS, IVIFS, and IVPyFS. The thought of PAOs provides the combination of the weights of attributes and reduces the chances of errors. Also, in AA operations, the changeability fact parameter gives a more versatile ground to the decision-maker for precise results. So, by using the advantages of PAOs, we proposed new AOs under AA operational rules. Finally, proposed AOs called IV-q-ROFAAPWA and IV-q-ROFAAPWG operators. We also demonstrate the basic properties of AOs, like boundedness, monotonicity, and idempotency. The MAGDM algorithm is constructed based on IV-q-ROFAAPWA and IV-q-ROFAAPWG operators and solves the real-life electric car selection problem. It is observed that by using the diagnosed IV-q-ROFAAPWA operator, the most suitable option is ${Ų}_{5}$ while by using the suggested concept of IV-q-ROFAAPWG operators, the most appropriate alternative is ${Ų}_{4}$. The influence of changing the value of parameters q and Ή is discussed, and the effect of ranking order is demonstrated in Table 10 and a graphical representation. It is also clearly noticed that AA operations cannot aggregate information when we place the parameter value as an even number while aggregating fuzzy details. It can only aggregate the data when we take the weight of the parameter as an odd value. To show the superiority of proposed IV-q-ROFAAPWA and IV-q-ROFAAPWG operators, we comprehensively compare existing AOs in different fuzzy environments. The visual representation of the comparative study is also part of the article.

Soon, we extend our proposed IV-q-ROFAAPWA and IV-q-ROFAAPWG approaches for the bipolar fuzzy framework given in Ref. [65] and Picture fuzzy Maclaurin symmetric AOs provided in Ref. [23]. The idea of spherical FS is presented in Ref. [66], and the thought of TSFS is delivered in Ref. [67]. The thought of prioritized AOs is based on IFS given in Ref. [68] and the theory diagnosed for complex bipolar fuzzy sets presented in Ref. [69]. Dombi AOs for complex q-ROFS are given in Ref. [70]. Some PAOs are based on the q-ROFS theory developed in Ref. [71] and the thought of Muirhead mean operators for t-spherical FS discussed in Ref. [72].

## Data availability statement

The datasets used and/or analysed during the current study available from the corresponding author on reasonable request.

## Funding

This research was funded by Philosophy and Social Sciences Planning Project of the 10.13039/100009950Ministry of Education grant number [21YJCZH203].

## CRediT authorship contribution statement

**Nan Zhang:** Writing – review & editing, Resources, Formal analysis. **Muhammad Rizwan Khan:** Writing – review & editing, Software, Investigation, Funding acquisition. **Kifayat Ullah:** Writing – review & editing, Software, Investigation, Formal analysis. **Muhammad Saad:** Writing – review & editing, Validation. **Shi Yin:** Writing – original draft, Resources, Data curation, Conceptualization.

## Declaration of competing interest

The authors declare that they have no known competing financial interests or personal relationships that could have appeared to influence the work reported in this paper.

## References

[1] Zadeh L.A. (1965). “Fuzzy sets,” Information and control.

[2] Atanassov K.T. (Aug. 1986). Intuitionistic fuzzy sets. Fuzzy Set Syst..

[3] Yager R.R. (2013). 2013 Joint IFSA World Congress and NAFIPS Annual Meeting.

[4] Yager R.R. (2016). Generalized orthopair fuzzy sets. IEEE Trans. Fuzzy Syst..

[5] Da Silva I.A., Bedregal B., Bedregal B., Santiago R.H.N. (2021). An interval-valued Atanassov's intuitionistic fuzzy multi-attribute group decision making method based on the best representation of the WA and OWA operators. Journal of Fuzzy Extension and Applications.

[6] Peng Y., Xiaohe L., Jianbo S. (2021). A multi-attribute group decision making method considering both the correlation coefficient and hesitancy degrees under interval-valued intuitionistic fuzzy environment. Appl. Soft Comput..

[7] Rahman K., Ali A., Abdullah S., Amin F. (2018). Approaches to multi-attribute group decision making based on induced interval-valued Pythagorean fuzzy Einstein aggregation operator. New Math. Nat. Comput..

[8] Zulqarnain R.M., Siddique I., Iampan A., Baleanu D. (2022). Aggregation operators for Interval-valued Pythagorean fuzzy soft set with their application to solve Multi-attribute group decision making problem. Comput. Model. Eng. Sci..

[9] Zhou Y., Yang G. (2022). A novel linguistic interval-valued Pythagorean fuzzy multi-attribute group decision-making for sustainable building materials selection. Sustainability.

[10] Akram M., Ramzan N., Deveci M. (2023).

[11] Wang J., Zhou Y. (2021). Multi-attribute group decision-making based on interval-valued q-Rung Orthopair fuzzy power generalized Maclaurin symmetric mean operator and its application in online education platform performance evaluation. Information.

[12] Zhang C., Li D., Kang X., Liang Y., Broumi S., Sangaiah A.K. (2020). Multi-attribute group decision making based on multigranulation probabilistic models with interval-valued neutrosophic information. Mathematics.

[13] Jin F., Ni Z., Chen H. (2016). Interval-valued hesitant fuzzy Einstein prioritized aggregation operators and their applications to multi-attribute group decision making. Soft Comput..

[14] Qin J., Liu X., Pedrycz W. (2016). Multi-attribute group decision making based on Choquet integral under interval-valued intuitionistic fuzzy environment. Int. J. Comput. Intell. Syst..

[15] Gurmani S.H., Chen H., Bai Y. (2023). Multi-attribute group decision-making model for selecting the most suitable construction company using the linguistic interval-valued T-spherical fuzzy TOPSIS method. Appl. Intell..

[16] Akram M., Martino A. (Jan. 2023). Multi-attribute group decision making based on T-spherical fuzzy soft rough average aggregation operators. Granul. Comput..

[17] Senapati T. (2022). Approaches to multi-attribute decision-making based on picture fuzzy Aczel–Alsina average aggregation operators. Comput. Appl. Math..

[18] Ullah K., Hussain A., Ahmad A., Ali Z. (Aug. 2022). Novel interval valued T-spherical fuzzy mclaurin symmetric mean operators and their applications in multi-attribute group decision making problems. Operations Research and Engineering Letters.

[19] Khan M.R., Wang H., Ullah K., Karamti H. (Jan. 2022). Construction material selection by using multi-attribute decision making based on q-rung orthopair fuzzy aczel–alsina aggregation operators. Appl. Sci..

[20] Liu S., Yu W., Chan F.T.S., Niu B. (Feb. 2021). A variable weight‐based hybrid approach for multi‐attribute group decision making under interval‐valued intuitionistic fuzzy sets. Int. J. Intell. Syst..

[21] Mu Z., Zeng S., Wang P. (2021). Novel approach to multi-attribute group decision-making based on interval-valued Pythagorean fuzzy power Maclaurin symmetric mean operator. Comput. Ind. Eng..

[22] Khan R., Ullah K., Pamucar D., Bari M. (2022). Performance measure using a multi-attribute decision-making approach based on complex T-spherical fuzzy power aggregation operators. Journal of Computational and Cognitive Engineering.

[23] Ullah K. (2021). Picture fuzzy maclaurin symmetric mean operators and their applications in solving multiattribute decision-making problems. Math. Probl Eng..

[24] Garg H., Ullah K., Mahmood T., Hassan N., Jan N. (2021). T-spherical fuzzy power aggregation operators and their applications in multi-attribute decision making. J. Ambient Intell. Hum. Comput..

[25] Ullah K., Hassan N., Mahmood T., Jan N., Hassan M. (2019). Evaluation of investment policy based on multi-attribute decision-making using interval valued T-spherical fuzzy aggregation operators. Symmetry.

[26] Feng F., Zhang C., Akram M., Zhang J. (Jul. 2023). Multiple attribute decision making based on probabilistic generalized orthopair fuzzy sets. Granul. Comput..

[27] Akram M., Zahid K., Kahraman C. (2023). A PROMETHEE based outranking approach for the construction of Fangcang shelter hospital using spherical fuzzy sets. Artif. Intell. Med..

[28] Akram M., Zahid K., Kahraman C. (2023). Integrated outranking techniques based on spherical fuzzy information for the digitalization of transportation system. Appl. Soft Comput..

[29] Duleba S., Moslem S. (2019). Examining Pareto optimality in analytic hierarchy process on real Data: an application in public transport service development. Expert Syst. Appl..

[30] Menger K. (1942). Statistical metrics. Proc. Natl. Acad. Sci. U.S.A..

[31] Aczél J., Alsina C. (1982). Characterizations of some classes of quasilinear functions with applications to triangular norms and to synthesizing judgements. Aequationes Math..

[32] Arora R., Abraham A., Cherukuri A.K., Melin P., Gandhi N. (2020). Intelligent Systems Design and Applications.

[33] Wang X. (2008). Fuzzy number intuitionistic fuzzy arithmetic aggregation operators. Int. J. Fuzzy Syst..

[34] Wang W., Liu X. (2011). Intuitionistic fuzzy geometric aggregation operators based on Einstein operations. Int. J. Intell. Syst..

[35] Khan M.R., Ullah K., Khan Q. (Feb. 2023). Multi-attribute decision-making using Archimedean aggregation operator in T-spherical fuzzy environment. Reports in Mechanical Engineering.

[36] Garg H. (2019). Intuitionistic fuzzy hamacher aggregation operators with entropy weight and their applications to multi-criteria decision-making problems. Iranian Journal of Science and Technology, Transactions of Electrical Engineering.

[37] Sarkar A., Biswas A. (2021). Dual hesitant q-rung orthopair fuzzy Dombi t-conorm and t-norm based Bonferroni mean operators for solving multicriteria group decision making problems. Int. J. Intell. Syst..

[38] Liu P. (2013). Some Hamacher aggregation operators based on the interval-valued intuitionistic fuzzy numbers and their application to group decision making. IEEE Trans. Fuzzy Syst..

[39] Wang L., Garg H., Li N. (2021). Pythagorean fuzzy interactive Hamacher power aggregation operators for assessment of express service quality with entropy weight. Soft Comput..

[40] Zhang X., Liu P., Wang Y. (Jan. 2015). Multiple attribute group decision making methods based on intuitionistic fuzzy frank power aggregation operators. J. Intell. Fuzzy Syst..

[41] Gayen S., Biswas A., Sarkar A., Senapati T., Moslem S. (2023).

[42] Hussain A., Ullah K., Senapati T., Moslem S. (Jul. 2023). Complex spherical fuzzy Aczel Alsina aggregation operators and their application in assessment of electric cars. Heliyon.

[43] Moslem S., Stević Ž., Tanackov I., Pilla F. (Jun. 2023). Sustainable development solutions of public transportation:An integrated IMF SWARA and Fuzzy Bonferroni operator. Sustain. Cities Soc..

[44] Moslem S. (2023). A novel parsimonious best worst method for evaluating travel mode choice. IEEE Access.

[45] Biswas T.K., Das M.C. (2019). Selection of commercially available electric vehicle using fuzzy AHP-MABAC. J. Inst. Eng.: Series C.

[46] Serap T. (2021). The interval-valued spherical fuzzy based methodology and its application to electric car selection. Düzce Üniversitesi Bilim ve Teknoloji Dergisi.

[47] Ziemba P. (2021). Multi-criteria approach to stochastic and fuzzy uncertainty in the selection of electric vehicles with high social acceptance. Expert Syst. Appl..

[48] Aminuddin A.S.A., Na’im Ku Khalif K.M., Jamil F.C., Jaini N.I. (2019). Journal of Physics: Conference Series.

[49] Guo S., Zhao H. (2015). Optimal site selection of electric vehicle charging station by using fuzzy TOPSIS based on sustainability perspective. Appl. Energy.

[50] Ghose D., Pradhan S., Tamuli P., Shabbiruddin (2019).

[51] Sharma K., Agrawal A., Bandopadhaya S., Roy S. (2019). 2019 *IEEE 5th International Conference for Convergence in Technology (I2CT)*.

[52] Gao H. (2016). The impact on the environment and economy due to the introduction of electric cars: based on the fuzzy synthetical evaluation method. J. Appl. Math. Phys..

[53] Taplin J. (2004). 26th Conference of Australian.

[54] Ghosh A. (2021). Application of hexagonal fuzzy MCDM methodology for site selection of electric vehicle charging station. Mathematics.

[55] Yager R.R. (Nov. 2001). The power average operator. IEEE Trans. Syst. Man Cybern. Syst. Hum..

[56] Klement E.P., Mesiar R., Pap E. (2000). Integration with respect to decomposable measures, based on a conditionally distributive semiring on the unit interval. Int. J. Uncertain. Fuzziness Knowledge-Based Syst..

[57] Senapati T., Martínez L., Chen G. (2022). Selection of appropriate global partner for companies using q-rung orthopair fuzzy aczel–alsina average aggregation operators. Int. J. Fuzzy Syst..

[58] Liu P., Wang P. (2018). Some q-rung orthopair fuzzy aggregation operators and their applications to multiple-attribute decision making. Int. J. Intell. Syst..

[59] Khan S.A.R., Mathew M., Dominic P.D.D., Umar M. (2021).

[60] Donyatalab Y., Farrokhizadeh E., Shishavan S.A.S., Seifi S.H. (2020). Intelligent and Fuzzy Techniques: Smart and Innovative Solutions: Proceedings of the INFUS 2020 Conference, Istanbul, Turkey.

[61] Wan B., Zhang X., Xiong M., Wang Z. (2022). Interval-valued q-rung orthopair fuzzy QUALIFLEX decision analysis method with Dombi operators. Discrete Dynam Nat. Soc..

[62] Chen S.-M., Lee L.-W., Liu H.-C., Yang S.-W. (2012). Multiattribute decision making based on interval-valued intuitionistic fuzzy values. Expert Syst. Appl..

[63] Senapati T., Chen G. (2021). Some novel interval-valued Pythagorean fuzzy aggregation operator based on Hamacher triangular norms and their application in MADM issues. Comput. Appl. Math..

[64] Alhamzi G., Javaid S., Shuaib U., Razaq A., Garg H., Razzaque A. (2023). Enhancing interval-valued pythagorean fuzzy decision-making through dombi-based aggregation operators. Symmetry.

[65] Mahmood T. (2020). A novel approach towards bipolar soft sets and their applications. J. Math..

[66] Mahmood T., Ullah K., Khan Q., Jan N. (2019). An approach toward decision-making and medical diagnosis problems using the concept of spherical fuzzy sets. Neural Comput. Appl..

[67] Ullah K., Garg H., Mahmood T., Jan N., Ali Z. (Feb. 2020). Correlation coefficients for T-spherical fuzzy sets and their applications in clustering and multi-attribute decision making. Soft Comput..

[68] Khan M.R., Raza A., Khan Q. (Dec. 2022). Multi-attribute decision-making by using intuitionistic Fuzzy rough Aczel-Alsina prioritize Aggregation Operator. Journal of Innovative Research in Mathematical and Computational Sciences.

[69] Mahmood T., ur Rehman U., Ali Z. (2023).

[70] Ali Z., Mahmood T. (2022). Some Dombi aggregation operators based on complex q-rung orthopair fuzzy sets and their application to multi-attribute decision making. Comput. Appl. Math..

[71] Multi-attribute Group Decision-Making Based on Q-Rung Orthopair Fuzzy Aczel–Alsina Power Aggregation Operators,” Engineering Applications of Artificial Intelligence, vol. vol. 126, p. 106629, Nov. 2023, doi: 10.1016/j.engappai.2023.106629.

[72] Akram M., Naz S., Feng F., Shafiq A. (May 2023). Assessment of hydropower plants in Pakistan: Muirhead mean-based 2-tuple linguistic T-spherical fuzzy model combining SWARA with COPRAS. Arabian J. Sci. Eng..

